# Specific Binding Agents for the Molecular Recognition of Biotoxins: Recent Advances and Applications in Forensic Toxicology

**DOI:** 10.3390/molecules31162786

**Published:** 2026-08-10

**Authors:** Congying Li, Wenyi Wu, Qing Huang, Yishun Huang

**Affiliations:** 1 College of Environment and Public Health, Xiamen Huaxia University, Xiamen 361024, China; licy@hxxy.edu.cn (C.L.);; 2 Institute of Analytical Technology and Smart Instruments, Xiamen Huaxia University, Xiamen 361024, China; 3 College of Chemistry, Fuzhou University, Fuzhou 350108, China

**Keywords:** biotoxins, forensic toxicology, aptamers, molecularly imprinted polymers (MIPs), antibodies, signal amplification

## Abstract

The precise detection and profiling of biotoxins are of paramount importance in analytical and forensic toxicology. Investigating these toxicants within highly chaotic background matrices—ranging from postmortem biological fluids to suspected poisoning vehicles such as complex dietary and environmental samples—requires robust recognition molecules capable of overcoming severe interference. This comprehensive review summarizes recent analytical developments in biotoxin detection, categorizing molecular recognition platforms into three primary types: immunological recognition (antibodies and recombinant derivatives), aptamer-based recognition, and entirely synthetic recognition (molecularly imprinted polymer, MIP). To meet the rigorous ultra-trace demands of medicolegal analysis, we further discuss the strategic integration of these recognition elements with powerful catalytic amplification cascades, highlighting the transition from natural biological enzymes to highly durable nanozymes and DNAzymes. By detailing recent structural optimizations and preparation strategies, this review critically evaluates the respective advantages, matrix tolerances, and limitations of each recognition mode when applied to diverse and challenging analytical samples. Finally, we provide a forward-looking perspective on the translational potential of these specific binding agents, emphasizing how computational rational design and portable integration will overcome practical bottlenecks in modern toxicological investigations.

## 1. Introduction

Biotoxins are naturally occurring toxic substances exhibiting considerable diversity, with complex structures and mechanisms of action. They affect the nervous, digestive, and immune systems, presenting hazards such as nephrotoxicity, hepatotoxicity, immunotoxicity, neurotoxicity, and dermal toxicity, thereby causing severe disruption to human metabolic processes and bodily functions [1]. In recent years, frequent incidents of biotoxin poisoning, whether accidental, occupational, or intentional, have heightened the awareness of their latent dangers in medicolegal contexts. Tracing the precise origins of such lethal exposures inherently requires the rigorous analysis of suspected poisoning vehicles, including environmental water sources and complex dietary matrices, which serve as critical physical evidence alongside human biological specimens. In forensic toxicology—the application of analytical chemistry to medicolegal investigations—tracing the precise origins of such lethal exposures inherently requires the rigorous analysis of suspected poisoning vehicles. Consequently, complex dietary matrices (e.g., contaminated foods and alcoholic beverages) and environmental water sources serve as critical physical evidence alongside human biological specimens. In these forensic contexts, investigators frequently encounter a distinct set of highly lethal molecules, such as botulinum neurotoxin in fatal foodborne outbreaks, saxitoxin in marine environments, or aflatoxins in contaminated crops. Given their potent toxicity and exceedingly low lethal doses, these toxicants pose a significant threat to public health and constitute a critical challenge in forensic investigations [2]. Confronted with these threats, establishing accurate, robust, and efficient methodologies for the specific recognition and profiling of biotoxins has become a paramount priority in analytical and forensic toxicology.

To effectively investigate biotoxin-related poisoning, early detection and legally defensible confirmation are essential. Conventional analytical methodologies predominantly rely on liquid chromatography-tandem mass spectrometry (LC-MS/MS). While establishing the absolute gold standard for medicolegal evidence, these instrumental techniques face a critical bottleneck: severe susceptibility to matrix effects (e.g., ion suppression) when analyzing highly chaotic forensic samples. In particular, investigating suspected poisoning vehicles presents severe analytical constraints: complex food extracts are heavily burdened with carbohydrates, dense lipids, and interfering proteins that frequently cause severe non-specific binding (biofouling) on sensor interfaces, while environmental samples often contain humic acids and variable salinity that can disrupt molecular interactions. Consequently, LC-MS/MS demands rigorous, highly selective sample pretreatment prior to analysis. Furthermore, these centralized instruments cannot fulfill the critical need for rapid, on-site triage during acute poisoning outbreaks. This dual challenge—the necessity for ultra-clean sample extraction to enable MS confirmation and the demand for portable, rapid screening—is precisely where specific binding agents become indispensable. By selectively isolating target biotoxins directly from complex background matrices, these recognition molecules empower both high-fidelity sample purification (e.g., affinity chromatography) and the development of rapid diagnostic biosensors. Therefore, advancing modern biotoxin analysis hinges on two pillars: the absolute specificity of the target recognition and the ultra-sensitivity of the signal output.

Addressing the first pillar, the arsenal of specific binding agents has evolved significantly to meet demanding analytical needs. While traditional immunoassay techniques utilizing natural antibodies laid the foundation for biotoxin quantification [3,4,5,6], the biological fragility of native proteins in harsh forensic extraction solvents has driven a paradigm shift toward more robust, engineered alternatives. Consequently, recombinant antibodies [7,8], nucleic acid aptamers (selected in vitro) [9,10,11,12,13,14,15,16], and entirely synthetic molecularly imprinted polymers (MIPs) [17] have emerged as powerful analytical tools. Their emergence is driven by three key advantages over natural antibodies: (i) superior stability in organic solvents and extreme pH conditions, which is critical for forensic sample preparation; (ii) reproducible synthesis or in vitro selection, circumventing batch-to-batch variability inherent to animal-derived antibodies; and (iii) rational tunability—MIPs can be imprinted against non-immunogenic or toxic targets, aptamers can be engineered with modified nucleotides for enhanced nuclease resistance, and recombinant antibodies can be reformatted as fragments (e.g., scFv, Fab) for improved tissue penetration or surface immobilization. These advanced platforms offer programmable affinities and exceptional physicochemical resilience against chaotic biological matrices. Transitioning to the second pillar, translating these nanoscale recognition events into highly measurable macroscopic readouts at trace levels requires profound signal amplification. To achieve this, specific binding agents are increasingly integrated with powerful catalytic cascade reactions [18,19]. While natural biological enzymes remain widely utilized, the field is rapidly pivoting toward highly stable synthetic analogs, namely nanozymes and DNAzymes, which offer unparalleled durability and enzyme-like turnover in extreme medicolegal environments.

**Figure 1:**
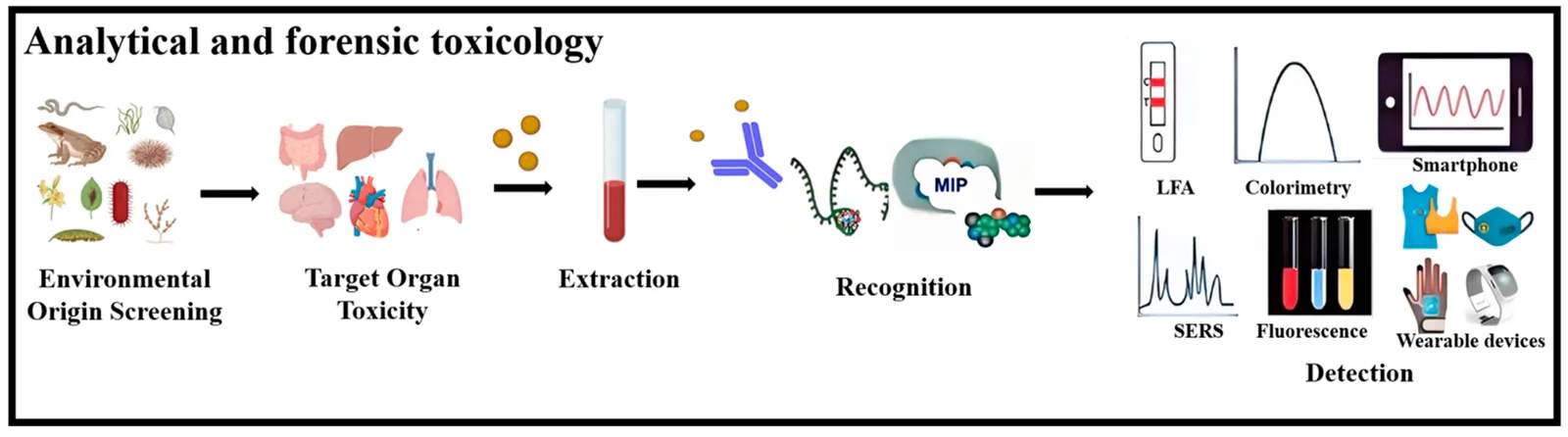
Schematic illustration of biotoxin recognition elements and their applications in forensic toxicology.

To accelerate the modernization of forensic toxicology, this review strategically highlights cutting-edge molecular recognition platforms emerging across broader analytical and biochemical fields (Figure 1). By examining analytical breakthroughs capable of isolating trace toxicants from profoundly complex background matrices (e.g., dietary, environmental, and clinical samples), we extrapolate their tremendous translational potential for addressing the rigorous demands of medicolegal investigations. Beginning with the fundamental principles of molecular recognition, we comprehensively detail the preparation strategies and structural evolution of specific binding agents, namely antibodies, aptamers, and MIPs. Furthermore, we critically evaluate recent analytical developments, comparing the respective advantages and limitations of each recognition platform when applied to complex medicolegal samples. To address the rigorous demands of trace-level analysis, we also dissect the strategic coupling of these recognition elements with cutting-edge catalytic amplification methodologies (enzymes, nanozymes, and DNAzymes). Ultimately, by illuminating the core structural and chemical logic behind these analytical innovations, this review aims to provide a robust, forward-looking reference for researchers addressing practical challenges in modern toxicological investigations.

## 2. Classification and Physicochemical Properties of Biotoxins

**Figure 2:**
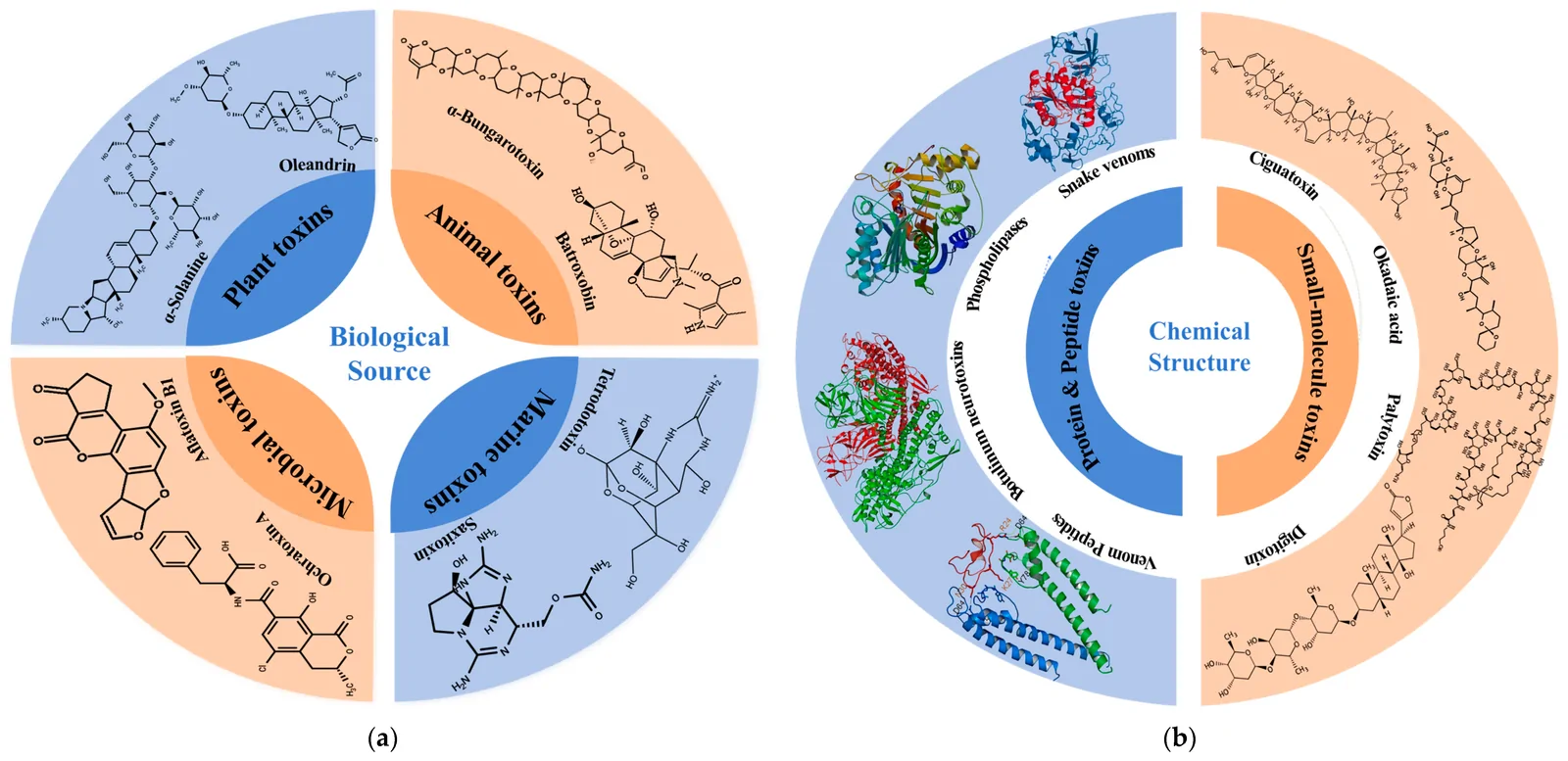
Classification of biotoxins. (**a**) Biological origin. (**b**) Chemical structure.

Biotoxins, commonly known as biological or natural toxins, are potent metabolic products synthesized by living organisms [20]. Exhibiting considerable structural diversity and complex mechanisms of action, these substances severely disrupt human metabolic processes and physiological functions, frequently culminating in acute poisoning or fatal outcomes [21]. In forensic toxicology, the accurate identification of these agents is paramount for determining the cause of death or investigating intentional poisonings. Because the physicochemical properties of a given biotoxin directly dictate its interaction with analytical matrices, these characteristics fundamentally guide the design and selection of specific binding agents. Generally, biotoxins are categorized along two primary dimensions: biological origin and chemical structure (Figure 2) [22].

### 2.1. Biotoxins Classified by Biological Source

Based on their biological origins, biotoxins are broadly classified into four categories: plant, animal, microbial, and marine toxins. Plant toxins possess diverse molecular architectures and are predominantly derived from families such as Solanaceae, Apocynaceae, and Fabaceae [23]. For instance, α-solanine, isolated primarily from Solanaceae, induces central nervous system toxicity via cholinesterase inhibition [24]. Animal toxins, prevalent in snake venom and venomous arthropods, critically disrupt nervous system functions and ion channel dynamics [25]. A notable example is α-cobratoxin, a neurotoxin that blocks neuromuscular transmission by competitively binding to nicotinic acetylcholine receptors (nAChR) [26]. Microbial toxins encompass bacterial toxins (exotoxins and endotoxins) and mycotoxins [27]. Aflatoxins, for example, are highly toxic and carcinogenic mycotoxins that present severe acute toxicity profiles [28,29]. Lastly, marine toxins—originating from microalgae, shellfish, and fish (e.g., Dinophyceae and Bacillariophyta)—can bioaccumulate in marine food webs, inflicting severe damage on the nervous, gastrointestinal, and cardiovascular systems upon accidental or intentional ingestion [30,31,32,33,34]. In forensic contexts, understanding these diverse biological origins is critical for tracking exposure pathways, identifying the precise causative agents in targeted poisonings, and investigating severe toxicological outbreaks.

### 2.2. Biotoxins Classified by Chemical Structure

From the perspective of molecular recognition and analytical chemistry, classifying biotoxins by chemical structure is arguably more pertinent. This approach primarily divides them into macromolecular (protein/peptide) toxins and small-molecule toxins. Protein and peptide toxins possess complex, three-dimensional macromolecular structures characterized by multiple spatial epitopes, with their toxicity typically driven by catalytic enzyme activity or membrane perturbation [35]. Their macromolecular nature generally renders them highly immunogenic, making them ideal targets for traditional antibody-based recognition. Conversely, small-molecule toxins possess low molecular weights, exhibit profound lethality at trace concentrations, and are rapidly absorbed and distributed within biological tissues. Because they intrinsically lack immunogenicity and are frequently present at minimal doses within complex medicolegal matrices (e.g., blood, urine, gastric contents), they are notoriously elusive to isolate and detect using conventional means [36,37]. Furthermore, in authentic forensic casework (e.g., analyzing urine or hepatic tissues), parent toxins are often rapidly biotransformed. Therefore, analytical methods and specific binding agents must frequently target their metabolized versions or covalent biomacromolecular adducts to accurately reconstruct exposure history. 

For aflatoxins, hepatic Cytochrome P450 enzymes biotransform AFB1 not only into the hydroxylated urine marker AFM1, but also into AFQ1 and AFP1, which represent major detoxified biliary and urinary metabolites. More importantly, the highly reactive intermediate AFB1-exo-8,9-epoxide covalently binds to cellular macromolecules, forming AFB1-N7-guanine adducts (which are subsequently excised and excreted in urine, reflecting acute DNA damage) and AFB1-albumin adducts in blood [28,29]. Due to the long half-life of serum albumin, the AFB1-albumin adduct serves as an invaluable long-term forensic biomarker of chronic exposure.

A similar requirement for metabolite-centric diagnostic recognition is observed across other toxin classes. Lipophilic marine polyethers like okadaic acid (OA) are frequently converted into acyl-esters (collectively referred to as DTX3) [38] via metabolic esterification in shellfish and digestive systems, requiring alkaline or enzymatic hydrolysis to liberate parent toxins prior to biosensing. Similarly, the highly lethal plant alkaloid aconitine undergoes rapid in vivo deacetylation to form benzoylaconine and aconine [39]. Consequently, engineering highly specific molecular recognition agents—particularly resilient synthetic platforms such as aptamers and MIPs—that effectively target and extract these low-molecular-weight chemical structures is a fundamental prerequisite for advancing modern forensic toxicology.

## 3. Strategies for Targeting Biotoxins

The vast structural diversity and potency of biotoxins inherently complicate their accurate identification. Furthermore, in toxicological investigations, these toxicants invariably occur at extremely low concentrations within complex medicolegal matrices (e.g., blood, urine, and postmortem tissues), where the detection process is frequently subject to severe background interference. In recent years, leveraging highly specific molecular recognition mechanisms has emerged as a crucial strategy to overcome these analytical challenges. Molecular recognition relies on the use of targeted binding agents that possess exceptional affinity and selectivity towards target analytes. Capitalizing on these bio-physicochemical interactions enables the precise differentiation of biotoxin analogues and the rapid quantification of trace-level toxicants directly from raw samples.

This comprehensive review categorizes these advanced targeting strategies into three primary domains: immunological recognition (antibodies and their derivatives), nucleic acid-based recognition (aptamers), and synthetic chemical recognition (MIPs). Each methodology is critically evaluated regarding its fundamental principles, structural optimizations, and practical forensic applicability, ultimately providing a forward-looking perspective on the evolutionary trajectory of biotoxin detection platforms.

### 3.1. Antibody Derivative Recognition Molecules

Antibodies and their structurally engineered derivatives remain the preeminent recognition elements in rapid analytical and toxicological assays. While the canonical antibody is a Y-shaped glycoprotein wherein the two upper Fab arms recognize specific antigens and the lower Fc tail mediates immune effector functions, this full-length structure is frequently modified for analytical sensing. To enhance targeting specificity and mitigate non-specific matrix interference, scientists have leveraged protein engineering to develop diverse antibody architectures, such as truncated fragments and mutated binding domains. Within the scope of biotoxin analysis, these protein- and peptide-based recognition elements are primarily categorized into four platforms: (I) polyclonal antibodies, (II) monoclonal antibodies, (III) recombinant antibodies, and (IV) synthetic affinity peptides [3,40]. By binding to target biotoxins with exceptional affinity, these recognition molecules enable the efficient extraction of toxicants from complex medicolegal matrices and facilitate precise quantification via downstream signal transduction. As summarized in Table 1, each platform presents distinct physicochemical advantages and analytical limitations. Consequently, in forensic practice, the selection of the most appropriate recognition platform is strictly dictated by the specific structural traits of the target biotoxin, the requisite detection limits, and the available laboratory infrastructure.

#### 3.1.1. Polyclonal Antibodies

Polyclonal antibodies are secreted by multiple B-cell clones and can recognize diverse epitopes on a single antigen. They are relatively inexpensive and straightforward to produce. The inherent cross-reactivity and strong signal amplification provided by polyclonal antibodies can be advantageous for the broad preliminary screening of biotoxin classes. Historically, their application peaked between 1980 and 2010. For instance, Clarke et al. successfully purified polyclonal antibodies against ochratoxin from hen egg yolks [42], while Chu et al. generated rabbit antisera against saxitoxin (STX) and neo-STX [43,44]. Similarly, polyclonal platforms targeting tetrodotoxin (TTX), yessotoxins, azaspiracid (AZA), and domoic acid (DA) have been established. Sato and Vlasenko independently developed anti-TTX polyclonal antibodies with enzyme-linked immunosorbent assay (ELISA) detection limits of 0.96 ng/mL and 25.54 ng/mL, respectively [45,46]. Demonstrating their utility in challenging matrices, an ELISA employing polyclonal sheep antibodies successfully quantified yessotoxins within highly complex biological matrices (e.g., shellfish tissue) with a limit of quantitation of 75 µg/kg. This assay exhibited the exceptional analytical sensitivity required to detect trace toxicant levels well below established regulatory thresholds, a capability that is highly translatable to trace forensic screening [47]. Expanding on analytical innovations, Samdal et al. reported a competitive ELISA integrated into an electrochemical detection system. This approach utilized protein G magnetic beads coated with polyclonal anti-AZA antibodies to efficiently extract and detect AZA analogues (AZA 1–10) [5]. Furthermore, addressing the critical need for high-throughput multiplexing, Maguire et al. integrated polyclonal antibodies targeting DA and STX—alongside a recombinant antibody against microcystin-LR (MC-LR)—onto a multi-channel microfluidic disc [48]. 

Overall, while polyclonal antibodies exhibit high overall avidity and serve as valuable tools for the broad preliminary screening of biotoxins, their forensic utility is fundamentally constrained by inherent biological limitations. Their poor batch-to-batch uniformity and relatively weak specificity render them highly prone to nonspecific cross-reactivity in complex immunological assays [49]. In medicolegal investigations, this lack of absolute specificity significantly increases the risk of false-positive results, often necessitating the use of more highly specific recognition molecules or downstream confirmatory mass spectrometry.

#### 3.1.2. Monoclonal Antibodies

Monoclonal antibodies (mAbs) are produced via classic hybridoma technology, originating from a single immortalized B-cell clone to target one specific antigenic epitope. They exhibit highly uniform physicochemical properties, homogeneous binding kinetics, and remarkable specificity in antigen binding. Furthermore, mAbs offer distinct analytical advantages, including high avidity to specific biotoxins (or structurally related biotoxin classes) and the capacity for unlimited in vitro proliferation. These characteristics render them indispensable for stringent forensic and analytical applications where extreme precision and absolute reproducibility are critical [50]. 

Following the development of the first mAb against aflatoxin B1 in 1983 [51], the application of mAbs targeting diverse biotoxins has proliferated exponentially. Based on these highly specific molecules, modern immunoassay methodologies now encompass the qualitative, semi-quantitative, and precise quantitative profiling of biotoxins. For instance, Zhou et al. employed a brevetoxin 2 (PbTx-2) hapten to prepare an mAb that presented low IC_50_ values of 6.40, 6.57, and 5.31 μg/kg for PbTx-2, PbTx-1, and PbTx-3, respectively, with a corresponding indirect competitive ELISA (icELISA) limit of detection (LOD) of 0.60 ng/well [52]. Expanding upon this, Zhang et al. subsequently established an icELISA using a broad-spectrum mAb to detect PbTx congeners in highly complex biological matrices (e.g., oyster tissue), achieving an LOD of 124.22 μg/kg and highlighting the robust anti-interference capabilities of mAbs [53]. Advancements in cell fusion techniques have further enhanced affinity; Wang et al. utilized Sp2/0 myeloma cell fusion with immunized mouse splenocytes to successfully isolate an mAb exhibiting exceptional affinity for okadaic acid (OA) [54]. Addressing the critical forensic challenge of trace-level toxicants, Tsumuraya et al. innovatively developed a highly sensitive fluorescent ELISA utilizing an immunoplate coated with two distinct mAbs, achieving the specific detection and quantitative analysis of four major ciguatoxin (CTX) congeners with an unprecedented LOD below 1 pg/mL [55]. 

Furthermore, mAbs are highly adaptable for rapid on-site testing. Zhang et al. reported a universal mAb capable of the simultaneous detection of aflatoxins B1, B2, G1, and G2 [56,57]. By incorporating this antibody into lateral flow test strips, they achieved a visual LOD of 0.46 ng/mL for total aflatoxins, demonstrating a rapid screening format highly applicable to initial stages of medicolegal investigations. 

Finally, the exceptional reproducibility of mAbs has been rigorously validated across multiple independent studies. Using an anti-TTX mAb, distinct research teams, including those of Kentaro, Campbell, and Reverté, conducted ELISA assays at different time points, consistently achieving virtually identical detection limits of approximately 2 ng/mL [58,59,60]. This conclusively demonstrates that the batch-to-batch variation in mAbs is significantly lower than that observed in polyclonal antibodies, ensuring the strict analytical reliability and legal defensibility required for forensic toxicology. Building on the exceptional reliability and specificity of these antibodies, several ELISA kits have been successfully commercialized for rapid biotoxin screening. For instance, the BÜHLMANN Amanitin ELISA kit [61] is widely validated for clinical and forensic diagnostics in human serum and urine. In the realm of biothreats, Tetracore offers highly sensitive BioThreat Alert kits for ricin and botulinum neurotoxins, which feature built-in background subtraction to mitigate false positives in highly degraded forensic matrices [62]. Furthermore, for marine and freshwater biotoxins, commercial platforms from manufacturers such as Abraxis and Beacon Analytical Systems provide broad-spectrum screening capabilities for targets like saxitoxin and microcystins [63]. These commercialized assays effectively bridge the gap between academic antibody development and real-world forensic applications.

#### 3.1.3. Recombinant Antibodies

Recombinant antibodies represent a significant evolutionary step beyond traditional antibody production. In this sophisticated genetic engineering approach, gene sequences encoding the variable heavy (V_H_) and light (V_L_) chain regions are cloned into expression vectors and subsequently introduced into host cells for scalable expression and purification [64]. Unlike polyclonal and monoclonal antibodies, recombinant platforms eliminate the need for continuous animal immunization, saving time and resources while vastly broadening the scope of in vitro antibody screening. By retaining the primary antigen-binding activity while shedding extraneous Fc structures, genetically engineered antibodies minimize nonspecific binding, which is a crucial advantage when analyzing complex biological matrices. Consequently, they offer broader and more robust application prospects in forensic and analytical toxicology than their natural counterparts [65,66]. In recent years, this technology has emerged as a rapidly advancing field, with the primary formats utilized in biotoxin detection including single-chain variable fragments (scFv), antigen-binding fragments (Fab), and nanobodies (Nb) [3].

An scFv is a compact recombinant protein engineered by genetically fusing the antibody V_H_ and V_L_ domains via a flexible peptide linker. It retains the parent antibody’s binding specificity while exhibiting enhanced tissue penetration, reduced immunogenicity, and vastly improved expression efficiency. Currently, scFv is the most prevalent recombinant format for detecting biotoxins. While early research frequently emphasized scFv-mediated therapeutic neutralization, its application in developing robust analytical sensors and sample extraction platforms remains a rapidly expanding frontier with substantial developmental potential for toxicology. For instance, He et al. purified antibody genes from hybridoma cells secreting an anti-OA monoclonal antibody and engineered an scFv in a V_H_-V_L_ format. This recombinant construct demonstrated superior sensitivity and specificity compared to the original monoclonal antibody, while significantly reducing preparation costs [67]. Employing a similar methodology, Liu et al. produced an scFv targeting MC-LR, achieving a highly sensitive detection limit of 0.3 ng/mL [68]. 

Phage display technology serves as the premier source for discovering novel scFvs. This technique involves incorporating vast scFv libraries, which are amplified from diverse B-cell repositories, into filamentous phages to establish natural or immune-phage display libraries. Following multiple rounds of biopanning (screening), scFvs with exceptional target affinity can be isolated. The efficacy of this methodology for toxicological screening has been extensively validated, with multiple research groups successfully assembling phage libraries derived from mice, rabbits, chickens, sheep, and humans. For example, Xu et al. assembled a *Bacillus thuringiensis* Cry1F toxin-immunized rabbit phage library to screen for highly active scFvs, yielding an IC_50_ value of 11.56 ng/mL and an icELISA detection limit of 0.18 ng/mL [69]. Similarly, Hu et al. constructed a phage-displayed mouse scFv against fumonisin B1 (FB_1_) exhibiting robust binding kinetics (K_D_ = 1.89 × 10^−7^ M) [70]. Highlighting the shift away from animal models, Sompunga et al. reported the de novo identification and characterization of anti-zearalenone (ZEN) scFvs directly from a naive human phage display library, offering a streamlined alternative to traditional recombinant production using hybridomas established from pre-immunized mice [71].

Antigen-binding fragments (Fabs) represent the specific regions of an antibody responsible for antigen recognition. Structurally, a Fab comprises the heavy chain’s variable (V_H_) and first constant (C_H1_) domains, paired with the entire light chain (V_L_ and C_L_ domains). Traditionally, Fabs were generated via the proteolytic digestion of animal-derived monoclonal antibodies. However, advancements in recombinant DNA technology and protein engineering now permit their direct in vitro expression, firmly establishing them within the class of recombinant antibodies [72]. While their low immunogenicity historically made them popular for the therapeutic neutralization of biotoxins, their robust structural stability is increasingly being leveraged for the precise analytical detection of toxicants in complex medicolegal matrices.

Highlighting their utility in toxicology, Nagumo et al. developed recombinant Fabs targeting CTXs utilizing phage display technology [73]. This approach achieved significantly more consistent production yields and structural stability compared to conventional enzymatic cleavage methods. Furthermore, Edupuganti et al. developed a highly sensitive immunoassay for aflatoxin B1 (AFB_1_) utilizing a recombinant Fab fragment [74]. This novel approach centered on a covalent Fab–AFB_1_ interaction, which increased detection sensitivity by nearly threefold, reduced assay duration, and facilitated antibody regeneration without compromising binding capacity, all of which are essential characteristics for cost-effective, high-throughput forensic screening. 

Despite these analytical advantages, the de novo generation of Fab fragments presents considerable technical challenges. Because assembling Fab phage libraries requires the precise co-expression and folding of both light and heavy chain fragments (totaling a molecular weight of approximately 50 kDa), attaining a highly diverse and functional library is inherently more complex than working with single-chain formats [75,76]. Consequently, Fab phage display libraries demand meticulous optimization and are generally more difficult to construct and handle than smaller antibody fragments separated from analogous libraries.

Nanobodies, formally classified as single-domain antibodies (sdAbs) or the variable domains of heavy-chain-only antibodies (VHH), are derived from naturally occurring unique antibody architectures. These are predominantly found as heavy-chain-only antibodies (hcAbs) in the serum of camelids, alongside analogous structural variants (IgNARs/V_NAR_) in cartilaginous fishes such as sharks, as fundamentally described by Hamers-Casterman et al. [77]. Representing the smallest known functional unit of antigen–antibody interaction (~15 kDa), these unique VHH domains are expressed as recombinant proteins. Compared to larger recombinant fragments like scFv and Fab, nanobodies possess equivalent or superior antigen-binding capacity, exceptional physicochemical stability, and an inherent ability to fold properly even in harsh microenvironments. This resilience, coupled with their ease of genetic modification, makes them exceptionally suited for biotoxin immunoassays in challenging medicolegal matrices [78].

Phage display technology remains the core methodology for screening high-affinity nanobodies. Recent research demonstrates that computationally assisted library design strategies can dramatically improve screening efficiency and target specificity. For example, the de novo construction of a synthetic phage display library featuring a rationally designed “glove-like cavity” enabled the successful isolation of high-affinity nanobodies targeting AFB_1_ [79]. Analogously, integrating rational library screening with ELISA formats achieved an ultra-low detection limit of 0.06 μg/L for MC-LR, showcasing the broad recognition capabilities of nanobodies across structurally diverse toxins [80]. 

Crucially for forensic toxicology, the advancement of dual-nanobody sandwich ELISA methodologies has resolved a persistent analytical bottleneck: the high incidence of false-positive results arising from endogenous matrix interference with the Fc-terminal region of conventional antibodies. Researchers established a secondary-antibody-free sandwich ELISA system utilizing highly specific nanobodies against α-haemolysin and *Staphylococcus aureus* enterotoxin B (SEB) [81,82]. This optimized architectural platform extended the linear detection range up to 512 ng/mL and achieved LODs of 10 ng/mL and 0.3 ng/mL, respectively. Subsequent engineering of multivalent (e.g., bivalent) nanobodies has further amplified overall avidity and detection sensitivity. Hughes et al. engineered bivalent nanobodies targeting SEB that remained structurally stable under high temperatures and within intricate biological matrices, enabling an LOD as low as 50 pg/mL [83]. Highlighting their utility in demanding forensic samples, Melli et al. reported a bivalent VHH-based immunoassay for Shiga toxin Stx2 utilizing a biotin–streptavidin amplification system. This assay exhibited quadruple the sensitivity of commercial ELISAs in highly complex fecal specimens, underscoring its practical applicability in toxicological diagnostics [84].

From a distinct analytical perspective, the utilization of anti-idiotypic nanobodies, which act as structural mimetics of the target antigens, has broadened the scope of competitive detection methodologies. Qiu et al. identified anti-idiotypic nanobodies against the Cry1Ab toxin via phage display biopanning, successfully developing a competitive immunoassay using these mimicked biotoxin epitopes [85]. Similarly, Shen et al. engineered nanobodies that mimic specific toxin domains to broadly recognize highly conserved receptor targets [86]. This structural mimicry offers a paradigm-shifting strategy for assay design, particularly when native biotoxin standards are exceptionally hazardous or difficult to isolate. Looking ahead, the integration of computational structural prediction with high-throughput selection techniques is anticipated to comprehensively advance the application of nanobodies in sophisticated forensic immunodiagnostics. 

#### 3.1.4. Affinity Peptides

Currently, synthetic affinity peptides serve as powerful alternative recognition elements to conventional antibodies, circumventing several inherent limitations of antibody-based platforms. Specifically, many low-molecular-weight biotoxins exhibit poor intrinsic immunogenicity, fundamentally complicating in vivo antibody generation. Furthermore, antibodies are encumbered by high production costs, susceptibility to denaturation in harsh extraction solvents, and large molecular weights (~150 kDa) that can induce steric hindrance on densely packed biosensor interfaces. In contrast, affinity peptides possess structurally well-defined, compact conformations capable of targeting specific functional groups on biotoxins via non-covalent interactions (e.g., van der Waals forces, hydrogen bonding, and electrostatic or hydrophobic interactions) [41]. Their primary analytical advantages lie in rapid in vitro solid-phase synthesis, straightforward site-directed modification (e.g., fusion with reporter labels), and exceptional structural resilience under extreme physicochemical conditions. Recent studies confirm that peptides function as highly robust recognition elements for biotoxin biosensors, in some cases demonstrating anti-interference capabilities that outperform natural antibodies in challenging analytical matrices [87,88,89,90,91,92,93]. 

The discovery and optimization of affinity peptides traditionally rely on high-throughput biological screening and combinatorial chemistry. Among biological methods, M13 filamentous phage display remains the most widely employed technique for targeting biotoxins [94]. Random peptide libraries, capable of displaying tens of millions of distinct epitopes, enable the rapid biopanning and isolation of high-affinity sequences. Complementing biological screening, advances in combinatorial chemistry allow peptides to be rationally synthesized and screened based on the target topology [95,96,97]. For example, Tozzi et al. utilized a combinatorial approach to isolate tetrapeptides exhibiting specific affinity for aflatoxins, marking the inaugural report of mycotoxin-binding peptides [98]. These selected sequences demonstrated robust binding constants ranging from 8.3 × 10^3^ M^−1^ to 1.2 × 10^4^ M^−1^, exhibiting selectivity functionally comparable to that of commercially available antibodies. Expanding upon combinatorial synthesis, Giraudi et al. developed a hexapeptide library via the one-bead-one-compound (OBOC) method, successfully identifying a specific sequence (SNLHPK) with highly favorable affinity for ochratoxin A (OTA) [99]. Notably, this peptide was utilized for the solid-phase extraction of OTA directly from wine—a complex, biochemically rich matrix replete with organic acids and alcohols—achieving a quantitative limit of detection as low as 0.10 μg/L. In forensic toxicology, complex dietary liquids like wine frequently serve as the primary poisoning vehicles (physical evidence) in intentional intoxication cases. The demonstrated ability to isolate toxicants from such chaotic matrices highlights profound translational potential for forensic sample preparation, such as extracting trace biotoxins from suspected beverages, highly degraded gastric contents or postmortem fluids.

Furthermore, advancements in computational rational design methodologies are fundamentally revolutionizing peptide discovery. Rather than relying solely on empirical library screening, peptides can now be precisely engineered through computer-aided structural design based on the strict molecular topology of the target biotoxin. Concomitantly, sophisticated molecular modeling and simulation (MMS) protocols are rapidly advancing to evaluate the binding thermodynamics and functionality of these engineered sequences in silico. Employing this exact methodology, Parker and Heurich, respectively, designed and expedited the assembly of peptide sequences targeting aflatoxin B1 (AFB_1_) and OTA, confirming their suitability for high-throughput detection through rigorous synthetic and analytical validation [100,101,102]. As the concluding class of antibody-derived recognition elements discussed herein, the integration of rationally engineered peptide biosensors holds profound promise for the future of forensic toxicology. By dramatically lowering assay development costs, prolonging the shelf-life of diagnostic platforms deployed in the field, and facilitating the high-throughput multiplexed profiling of trace toxicants, affinity peptides represent a critical transition toward next-generation medicolegal diagnostics [103]. 

### 3.2. Aptamers as Recognition Molecules

Aptamers are short, single-stranded oligonucleotide (≤100 nucleotides in length) sequences isolated in vitro through SELEX [104,105]. Because they are synthesized entirely via chemical pathways without the need for an immune-competent host organism, they are frequently termed “chemical antibodies.” Compared to traditional protein antibodies, aptamers present several profound analytical advantages, making them particularly formidable tools for forensic toxicology: (1) Broad Target Range: They can target an exceptionally diverse array of analytes, encompassing minute low-molecular-weight toxicants (which often lack intrinsic immunogenicity), macromolecules such as enzymes, and even entire pathogenic cells. (2) Exquisite Specificity: Upon target interaction, aptamers undergo adaptive folding. Through hydrogen bonding, electrostatic interactions, and van der Waals forces, they form specialized three-dimensional conformations (e.g., hairpin loops, stem-loops, and G-quadruplexes) that enable the precise recognition of minute structural variations among biotoxin congeners. (3) High Affinity: They exhibit robust binding capacities, with dissociation constants typically ranging from the micromolar down to the picomolar level. (4) Compact Size: Their low molecular weight allows access to cryptic target epitopes inaccessible to bulky immunoglobulins and minimizes steric hindrance on biosensor interfaces. (5) Exceptional Stability: Unlike easily denatured proteins, oligonucleotide fragments undergo reversible denaturation and renaturation. This resilience is critical for medicolegal applications, allowing aptamers to withstand the harsh solvents and extreme pH conditions often required for biotoxin extraction from degraded biological matrices. Furthermore, they can be preserved as dry powders for years without loss of function. (6) Rapid Discovery and Reproducible Production: The SELEX screening cycle is relatively short (typically 2–3 months), and their subsequent mass production relies entirely on automated solid-phase chemical synthesis. This fundamentally eliminates the batch-to-batch variability inherent in biological antibody production. (7) Facile Modification: They can be easily conjugated with diverse reporter labels, functional groups, or nanomaterials without compromising their target affinity [106,107]. 

To date, dozens of highly specific biotoxin aptamers have been successfully acquired through SELEX technology, as detailed in Table 2. Leveraging these programmable sequences, aptamer-based biosensors (aptasensors) are garnering intense scholarly focus, demonstrating immense potential for robust trace analysis. This section aims to provide a comprehensive overview of the current research landscape regarding biotoxin aptamers. By critically elucidating the recent advancements and inherent limitations of various SELEX methodologies, we intend to provide strategic guidance for engineering high-performance aptasensors tailored for complex medicolegal and toxicological investigations.

#### 3.2.1. Overview of Biotoxin Aptamer Screening Methods

**Figure 3:**
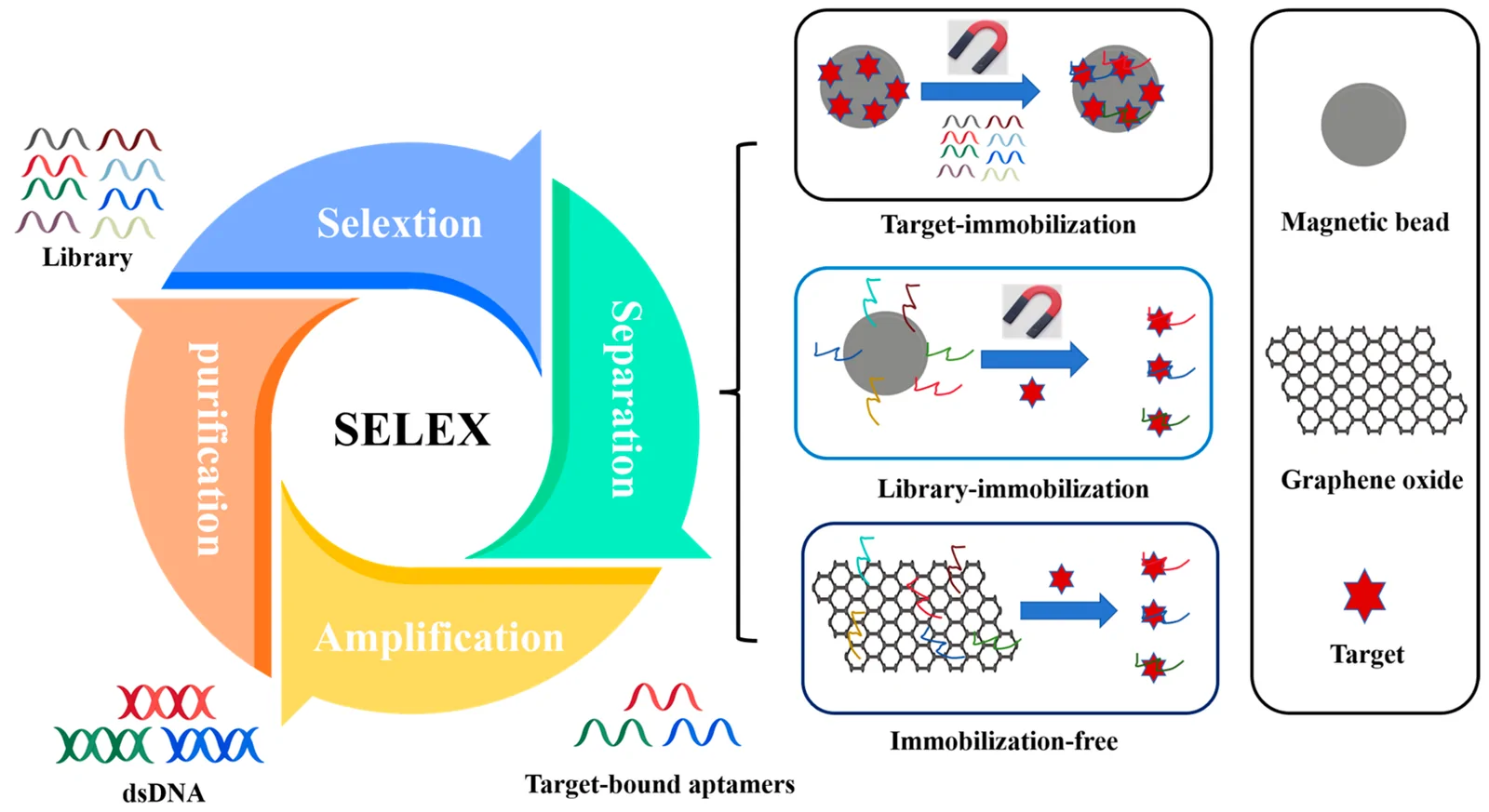
An illustration of the key steps of a typical SELEX protocol. Three different bound/unbound sequence separation methods are included. Created by the authors based on Ref. [107].

First established in 1990, the SELEX technique remains the gold standard for aptamer discovery [104,105]. The procedure typically commences by incubating the target biotoxin with a vast combinatorial library comprising 10^12^ to 10^15^ random single-stranded DNA (ssDNA) or RNA oligonucleotides. Following incubation, sequences capable of binding to the target molecule are partitioned from unbound sequences, eluted, and amplified via polymerase chain reaction (PCR) to form an enriched pool for the subsequent selection round. After multiple iterative cycles, the highly enriched oligonucleotide pool undergoes high-throughput sequencing and structural analysis. Since its inception, numerous modified SELEX protocols have been developed to isolate biotoxin aptamers, with the primary methodological differences centering on the strategies utilized to partition bound from unbound oligonucleotides [107]. As illustrated in Figure 3, this section classifies these methodologies into three primary categories: target-immobilization SELEX, library-immobilization SELEX, and immobilization-free SELEX.

Target-immobilization SELEX represents a classical partitioning strategy wherein the target biotoxins are covalently tethered to solid-phase supports—such as magnetic beads, agarose microspheres, affinity columns, or biolayers—prior to library incubation. This approach has successfully yielded robust aptamers for a variety of critical toxicants, including AFM_1_ [125], FB_1_ [115], OTA [112], PAT [121] and ZEN [126]. Because solid-phase immobilization facilitates the stringent washing and robust physical removal of non-specifically bound sequences, it remains highly suitable for diverse analytical applications [108,127,128,129]. 

**Figure 4:**
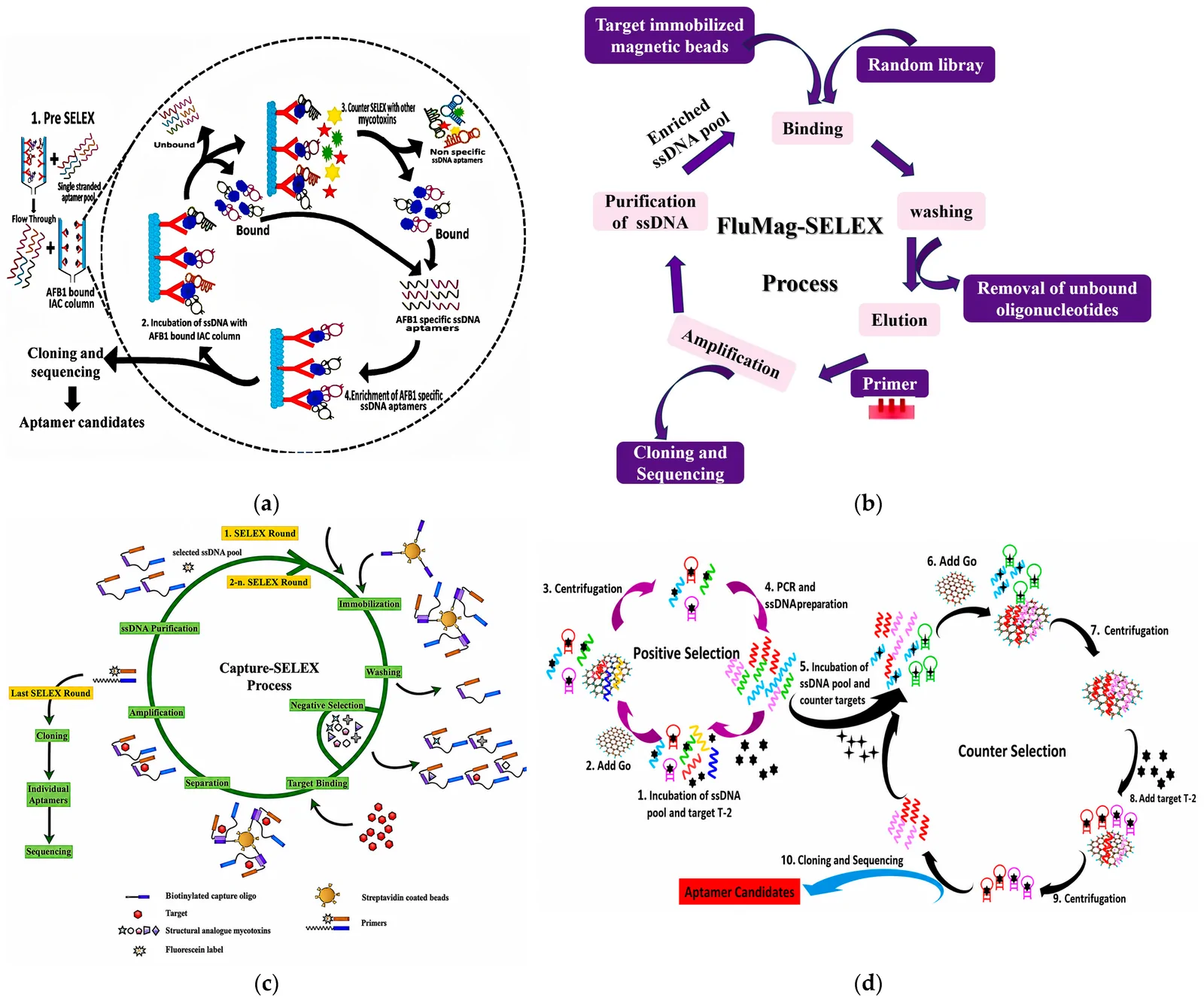
Schematic diagram of biotoxin aptamer selection. (**a**) Affinity columns-SELEX. Reprinted with permission from Ref. [108]. Copyright 2016, Frontiers Media SA. (**b**) FluMag-SELEX. Created by the authors based on Ref. [130]. (**c**) Capture-SELEX. Reprinted with permission from Ref. [135]. Copyright 2018, American Chemical Society. (**d**) GO-SELEX. Reprinted with permission from Ref. [124]. Copyright 2014, American Chemical Society.

Within this category, affinity column-SELEX offers exceptionally high separation efficiency. For instance, Setlem et al. employed an affinity column platform to isolate nanomolar-affinity aptamers for AFB_1_, as illustrated in Figure 4a, thereby substantially enhancing the final binding capacity of the sequences [108]. However, affinity columns often suffer from high non-specific retention on the matrix backbone itself. Furthermore, oligonucleotides exhibiting the strongest target affinity are notoriously challenging to elute, which can prolong the selection cycle and complicate sequence recovery.

To mitigate matrix retention and streamline processing, magnetic bead SELEX (Mag-SELEX) has emerged as the most widely employed target-immobilization technique to date. It is characterized by its straightforward operation, rapid magnetic separation, and adaptability to a broad spectrum of target molecules. Building on these principles, Stoltenberg et al. developed a fluorescently labeled variant (FluMag-SELEX) to monitor enrichment progress in real time (Figure 4b) [130]. Applying Mag-SELEX, researchers have successfully acquired high-affinity aptamers for various toxicological targets; for example, McKeague et al. isolated distinct aptamers for FB_1_ [115] and OTA [114], while Barthlemébs et al. [113] and Chen et al. [126] acquired highly specific aptamers for OTA and ZEN, respectively. These successful isolations are typically required between 14 and 15 screening rounds.

Library-immobilization SELEX reverses the traditional partitioning paradigm by immobilizing the oligonucleotide library itself onto solid substrates (e.g., magnetic or agarose beads) via complementary capture strands, allowing the target biotoxins to interact freely in solution. Upon encountering the target, highly specific sequences undergo target-induced conformational changes, triggering their dissociation from the complementary capture strand and subsequent release into the supernatant. This target-bound ssDNA is then recovered via magnetic separation or centrifugation. Over the past several years, the Wang research group has successfully utilized this approach—commonly termed Capture-SELEX—to isolate multiple high-affinity aptamers for advanced toxicological analysis [131,132,133,134]. Demonstrating its efficacy, Zhang et al. obtained an anti-ZEN aptamer with a highly favorable dissociation constant (K_D_ = 15.2 ± 3.4 nmol/L) after only eight screening rounds (Figure 4c) [135]. The paramount advantage of Capture-SELEX is that it entirely avoids the chemical modification or immobilization of biotoxin. This eliminates spatially induced steric hindrance and fully exposes the native binding epitopes of the target, ensuring that the selected aptamers possess optimal affinity for the “free” toxins typically encountered in liquid medicolegal matrices. However, immobilizing the library inherently restricts the spatial freedom of the oligonucleotides, which can reduce the effective sequence diversity and potentially exclude high-affinity candidates during the initial selection rounds. Furthermore, the base-pairing interactions used for library immobilization undergo a dynamic dissociation equilibrium, which can result in background leakage and complicate the screening procedures. Consequently, these technical limitations have resulted in a relatively restricted application of Capture-SELEX for biotoxin screening compared to target-immobilization methods.

Immobilization-free methodologies circumvent the myriad complications associated with solid-phase tethering. In the realm of small-molecule toxicant screening, Graphene Oxide SELEX (GO-SELEX) has emerged as the most prevalent immobilization-free platform [136,137,138,139]. GO-SELEX leverages the unique physicochemical properties of graphene oxide to partition sequences: free ssDNA or RNA inherently adsorbs onto the GO surface via strong π-π stacking interactions. However, upon binding to the target biotoxin, specific sequences fold into rigid three-dimensional conformations, disrupting the π–π stacking and causing them to spontaneously desorb into the solution. The desorbed, target-bound sequences are subsequently amplified and advanced to the next screening round. Demonstrating the robust applicability of this method, researchers successfully employed this principle to isolate aptamers against the T-2 toxin (Figure 4d) [124]. Subsequently, highly specific aptamers targeting patulin (PAT) [12] and STX [122] have also been generated via this route. 

While GO-SELEX is operationally streamlined and effectively preserves the native state of the target, its application is strictly constrained by the chemical nature of the biotoxin. Specifically, if a target biotoxin possesses highly aromatic structural motifs, it may inherently adsorb onto the GO surface, fundamentally nullifying the separation mechanism and severely limiting the target range. Furthermore, screening efficiency is highly dependent on library strand length [137]. Shorter nucleic acid chains exhibit weaker baseline adsorption to GO, rendering them prone to spontaneous self-desorption and elevated false-positive rates. Conversely, longer chains bind to GO with such avidity that target-induced desorption becomes thermodynamically unfavorable, severely hindering the recovery of genuine aptamers. Therefore, precisely optimizing the nucleotide sequence length within the starting library is an absolute prerequisite for ensuring the success of GO-SELEX in complex toxicological applications.

#### 3.2.2. Application of Aptamers in Biotoxin Assays

Over the past three decades, unprecedented progress has been made in translating biotoxin aptamers into functional aptasensors. By circumventing the need for elaborate sample purification protocols, these devices can deliver reliable results within 30 min. In forensic toxicology, this rapid turnaround is invaluable for on-site point-of-care testing, the mass screening of acute poisonings, and swift medicolegal diagnosis [140]. To meet the ultra-sensitive detection limits required for trace forensic evidence, diverse nucleic acid signal amplification strategies have emerged. These encompass enzyme-assisted techniques, such as DNA polymerase-driven rolling circle amplification (RCA), and enzyme-free methodologies, notably hybridization chain reaction (HCR) and catalytic hairpin assembly (CHA). For instance, Aflatoxin M1 (AFM1)—the major hydroxylated metabolized version of Aflatoxin B1—serves as a critical forensic biomarker excreted in biological fluids and milk following toxic exposure. A highly sensitive aptasensor for detecting this specific metabolite integrated time-resolved fluorescent nanoparticles with RCA technology to achieve a remarkable LOD of 0.0194 pg/mL in milk (a common dietary poisoning vehicle), surpassing previously published benchmarks [141]. Similarly, Zhao et al. designed an STX aptasensor combining gold nanozymes with aptamer-triggered HCR [142]. By catalyzing the TMB-H_2_O_2_ oxidation reaction, this assay achieved an LOD of 42.46 pM in complex biological matrices (e.g., authentic scallop extracts), demonstrating its robust anti-interference capabilities. 

Moreover, nuclease-assisted signal amplification methods—employing enzymes such as deoxyribonuclease I (DNase I), ribonuclease H (RNase H), exonuclease I (Exo I), and exonuclease III (Exo III)—have demonstrated immense analytical potential. Exploiting the selective degradability of specific dsDNA or ssDNA substrates by these nucleases, researchers have developed robust “target recycling” amplification strategies. In these highly efficient biological cascade reactions, a single biotoxin molecule can repeatedly trigger the release of signal probes [143]. For example, Xie et al. constructed an aptasensor for MC-LR utilizing FAM-labeled aptamers, gold nanoparticles, and DNase I [144]. In the presence of MC-LR, the aptamer undergoes a conformational shift, binding the toxin and dissociating from the fluorescence-quenching gold nanoparticle surface. Upon the addition of DNase I, the target-bound aptamer is selectively cleaved. Crucially, this cleavage releases the intact MC-LR back into the sample pool to bind another aptamer, initiating a continuous target-recycling cycle that yields intense fluorescence. This strategy not only dramatically amplifies the optical signal but also provides novel kinetic insights into dynamic chemical reactions at the single-molecule level.

Additionally, intelligent synthetic DNA systems leveraging DNA walkers and CRISPR-Cas machinery have revolutionized aptasensor programming. Specifically, the highly directional mechanical motion of DNA walkers enables repetitive signal cleavage; this motion is initially pre-locked by the aptamer and exclusively activated upon biotoxin binding [145]. Concurrently, CRISPR-Cas-based sensing systems utilize aptamers as highly specific activation triggers. While traditional enzymatic amplification can sometimes be hindered by the instability of enzymes in the demanding, inhibitor-rich environments of forensic matrices, the target-triggered *trans*-cleavage activity of CRISPR-Cas nucleases offers unparalleled specificity and efficiency [146]. For example, Mao et al. developed an innovative aptasensor for OTA employing CRISPR/Cas12a technology [147]. Upon OTA recognition, the sensor releases an activator cDNA strand that hybridizes with crRNA, triggering the Cas12a collateral cleavage of UCNP-DNA linkers attached to magnetic Fe_3_O_4_ nanoparticles. This cleavage activates a highly specific fluorescent signal, achieving an impressive LOD of 0.83 ng/mL. Moving forward, the integration of programmable CRISPR-Cas strategies—alongside highly durable enzyme-free circuits like HCR—holds tremendous promise for the robust, multiplexed detection of biotoxins in challenging medicolegal investigations.

### 3.3. Molecularly Imprinted Polymers as Recognition Molecules

Molecular imprinting technology (MIT) has emerged as a highly robust synthetic strategy for the specific recognition of biotoxins. By offering exceptional specificity, physicochemical stability, and reusability, MIT effectively circumvents the ethical and logistical constraints associated with in vivo animal immunization and biological antibody production [148]. Molecularly imprinted polymers (MIPs) generated via MIT are synthetic, highly crosslinked porous materials designed to exhibit outstanding target recognition properties. Compared to biological receptors such as antibodies and aptamers, MIPs provide distinct analytical advantages: extreme tolerance to temperature fluctuations, remarkable stability across diverse pH ranges, straightforward and cost-effective scalable synthesis, rapid responsiveness, and an extended shelf life. Over the past decade, both the publication volume and citation impact concerning the application of MIPs for the selective extraction and detection of biotoxins have expanded exponentially [149]. This section comprehensively reviews the fundamental principles of MIT and established synthetic methodologies for preparing MIPs tailored for demanding toxicological analyses.

#### 3.3.1. Principles of MIT

**Figure 5:**
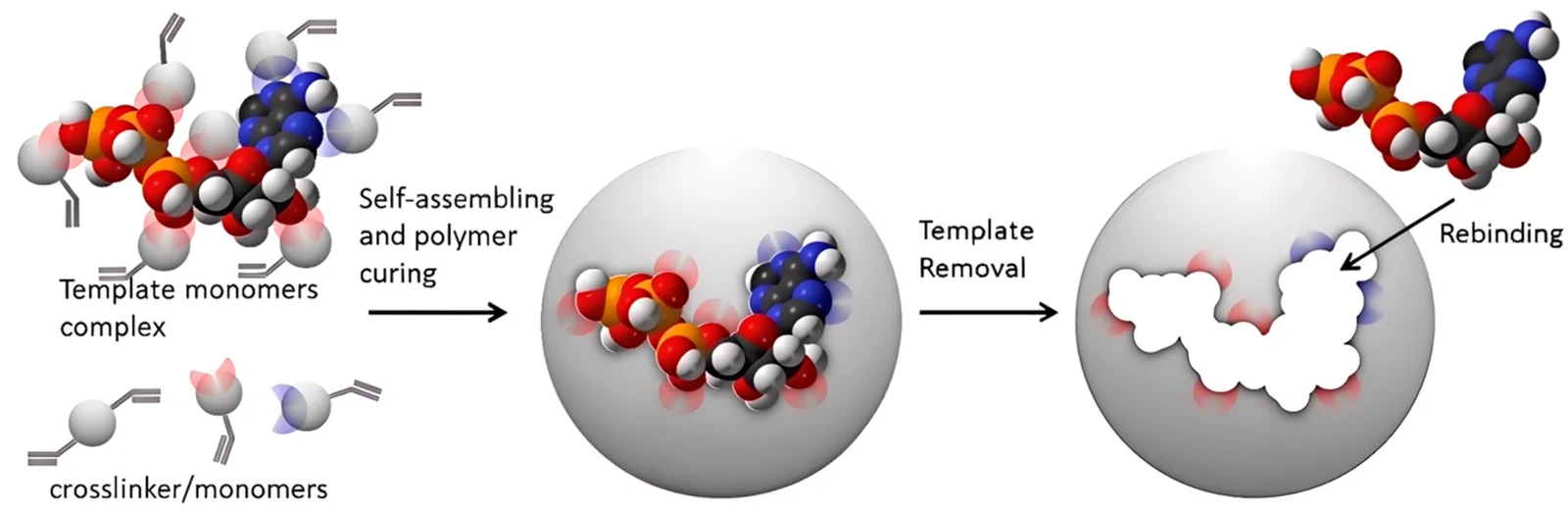
Fundamental principles of molecular imprinting techniques. Reprinted with permission from Ref. [150]. Copyright 2016, American Chemical Society.

Often conceptualized as a synthetic “lock-and-key” paradigm, MIT (also referred to as molecular templating technology) is a technique for engineering synthetic polymers with bespoke spatial cavities and binding sites that precisely match a target molecule. The synthesis of typical MIPs requires five essential components: a template molecule (the native biotoxin or a structural analog), functional monomers, a cross-linking agent, a polymerization initiator, and a porogenic solvent. The fundamental preparation principle is illustrated in Figure 5 [150]. Initially, the functional monomers and template molecules undergo a pre-polymerization assembly within an appropriate solvent, interacting via covalent or non-covalent forces to form a stable monomer-template complex. Subsequently, upon the addition of a cross-linker and activation by the initiator, a polymerization reaction proceeds to encase the complex within a rigid, highly crosslinked polymeric network. Finally, the template molecules are meticulously extracted (typically via extensive solvent washing), leaving behind three-dimensional nanocavities. These imprinted binding sites are perfectly complementary to the original template in shape, size, spatial arrangement, and functional group distribution. Consequently, the resulting MIPs can selectively and reliably rebind the target biotoxins, demonstrating exquisite molecular recognition even amidst complex interfering compounds [151,152,153].

To serve effectively in forensic and analytical toxicology, an optimal MIP must possess three critical properties: (1) high chemical and physical resilience to harsh sample preparation conditions (e.g., strong organic solvents, extreme pH, and elevated temperatures commonly required for extracting toxicants from biological tissues), alongside excellent reusability; (2) a straightforward synthesis protocol with low production costs; and (3) exceptional affinity, specificity, and selectivity toward the target analyte against severe background matrix interference. Capitalizing on these robust attributes, MIPs have been extensively applied for the isolation and identification of both macromolecular and small-molecule targets. In particular, they demonstrate outstanding performance as solid-phase extraction (SPE) sorbents and sensor recognition elements for isolating elusive toxicants—including illicit drugs, biological toxins, and lethal forensic biomarkers—directly from highly complex medicolegal matrices such as blood, urine, and postmortem fluids.

#### 3.3.2. Preparation of MIPs for Biotoxins 

MIPs targeting biotoxins are synthesized via diverse polymerization strategies tailored to optimize molecular recognition and sample extraction. Conventional methodologies primarily include bulk, suspension, emulsion, and precipitation polymerization [154,155,156,157]. Recently, the convergence of multidisciplinary fields has catalyzed the emergence of advanced synthetic strategies, such as surface imprinting, nano-polymerization, and click chemistry-mediated polymerization. Furthermore, the integration of dummy templates (virtual imprinting) and in silico computational modeling has revolutionized rational MIP design [158,159,160]. Table 3 summarizes the synthetic methodologies and physicochemical characteristics of these diverse MIP formats. 

The selection of an optimal polymerization technique is strictly dictated by the structural characteristics of the target biotoxin and the intended forensic or analytical application. For instance, MIPs utilized as SPE sorbents for routine sample pretreatment often employ bulk polymerization due to its high recognition efficiency and synthetic scalability. Bayram et al. synthesized multi-template MIPs via bulk polymerization using four aflatoxins (B_1_, B_2_, G_1_, and G_2_), leveraging these robust monolithic polymers to separate and enrich trace toxicants from highly complex matrices [171]. While these MIP applications are highly effective for extracting parent mycotoxins from suspected poisoning vehicles (e.g., food evidence or gastric contents), a critical future direction in forensic toxicology must focus on utilizing structural analogs of their biotransformed metabolites (e.g., synthesizing MIPs specifically imprinted against Aflatoxin M1) to enable robust solid-phase extraction directly from postmortem urine or clinical fluids. However, bulk polymerization typically yields solid monoliths that require extensive mechanical grinding and sieving. This post-processing can partially destroy spatial binding cavities and result in irregular particle sizes, potentially compromising chromatographic performance.

Conversely, emulsion and precipitation polymerizations generate uniform, sub-micron spherical particles. These formats offer highly accessible surface binding sites and superior mass transfer kinetics, rendering them ideal for sensor coatings and advanced chromatographic stationary phases. Jayasinghe et al. employed an azobisisobutyronitrile-initiated emulsion polymerization to construct a molecularly imprinted Mn: ZnS phosphorescent sensor. While initially validated in complex biological feeds, its robust linear quenching (2–20 μg/L) and extreme sensitivity demonstrate a high potential for adapting such optical sensors to trace toxin detection in forensic bodily fluids [172]. To further expedite sample preparation, Huang et al. synthesized novel magnetic MIPs using an Fe_3_O_4_ core and a virtual template (warfarin sodium) via precipitation polymerization [173]. Under an external magnetic field, these MIPs achieved the rapid isolation of the target analyte within a mere 100 s. Demonstrating a high adsorption capacity (7 mg/g) and an imprinting factor of 2.97, this magnetic separation strategy significantly reduces pretreatment time, a critical advantage in acute medicolegal investigations. Furthermore, surface polymerization technology directly onto transducer surfaces facilitates ultra-fast analyte contact and minimizes template diffusion constraints. Jiang et al. modified gold electrodes with p-aminothiophenol, utilizing electropolymerization to create ultra-thin, highly sensitive AFB_1_-imprinted films for linear sweep voltammetric detection [174].

Synthesizing biotoxin-imprinted polymers poses a unique occupational and analytical hazard: target biotoxins are often exceptionally lethal, and analytical samples frequently comprise intricate, heterogeneous matrices (e.g., antemortem and postmortem fluids) replete with interfering compounds. More critically, the incomplete removal of the actual biotoxin during MIP synthesis can lead to “template bleeding”, the gradual leakage of residual template molecules during actual sample analysis, which causes catastrophic false-positive results in trace forensic investigations. To elegantly circumvent these issues, virtual imprinting (dummy template) technology substitutes the highly hazardous target molecule with a safe, structurally analogous compound during the polymerization phase. Wang et al. achieved significant breakthroughs utilizing this technique, substituting hazardous targets with safer alternatives without compromising the structural fidelity or molecular recognition effectiveness of the resulting cavities [175]. Similarly, Zhao et al. employed hydroxypyridine and 6-hydroxynicotinic acid as complementary dummy templates to synthesize MIPs. Exhibiting exceptional selectivity and strong adsorption capacity for patulin, this contamination-free methodology offers robust advantages for extracting toxicants from complex clinical and diagnostic samples [176]. 

In recent years, the integration of computational modeling has substantially elevated the rational design and thermodynamic optimization of MIPs. Density Functional Theory (DFT), a quantum mechanics-based computational methodology, facilitates the quantitative theoretical characterization of micro-adsorption mechanisms between biotoxins and functional monomers at the atomic scale [160]. This in silico approach not only elucidates the precise non-covalent interaction energies but also provides predictive visualization of dynamic molecular conformations [177]. For example, Yang et al. developed an Independent Gradient Model combined with Electrostatic Potential Mapping (derived from Hirshfeld partitioning) to theoretically map the hydrogen bonding and van der Waals interactions at the polymer level [178]. Although effectively applied to extract ZEN from complex botanical matrices, such rigorous quantum mechanical screening allows analytical chemists to preemptively select the most effective functional monomers and cross-linkers before initiating physical synthesis. Ultimately, implementing these computational approaches in MIP design presents a highly sustainable, targeted pathway that minimizes hazardous laboratory waste, reduces reliance on dangerous chemical trials, and accelerates the development of bespoke sensors for forensic toxicology [179,180].

## 4. Catalytic Reactions for Signal Amplification in Molecular Recognition

In forensic toxicology, the accurate quantification of trace-level biotoxins within complex medicolegal matrices often demands sensitivities that exceed the baseline capabilities of unamplified molecular recognition. To overcome severe matrix interference and meet these stringent analytical requirements, the strategic integration of specific binding agents with enzyme-mediated catalytic reactions has become a pivotal strategy. Leveraging the exceptional turnover rates and substrate specificity of catalytic elements enables profound signal amplification, thereby converting a singular target-binding event into a highly measurable macroscopic signal. Currently, three primary classes of catalytic amplifiers are employed in biotoxin analysis: natural biological enzymes, synthetic nanozymes, and DNAzymes. This section evaluates these signal amplification strategies and their applicability to trace toxicological investigations. It is worth noting that while the majority of current catalytic biosensors are designed against parent mycotoxins (e.g., AFB1, OTA) frequently recovered from suspected food vehicles, these versatile amplification cascade strategies can be readily adapted to target their specific biotransformed metabolites in human bodily fluids, provided that the upstream specific binding agents (e.g., aptamers or nanobodies) are re-engineered for the specific metabolite.

### 4.1. Biological Enzymes

Natural biological enzymes boast exceptional catalytic activity and substrate specificity, rendering them traditional mainstays as signal amplification elements in analytical assays. The most extensively utilized classes in biotoxin detection include peroxidases, oxidases, and alkaline phosphatases (ALP). 

Peroxidases classically catalyze the oxidation of substrates via hydrogen peroxide (H_2_O_2_) to generate visually discernible colorimetric, fluorometric, or chemiluminescent signals. For instance, Gao et al. deployed horseradish peroxidase (HRP)-labeled aptamers as biosensing receptors targeting PTX immobilized on a sensor surface [181]. The localized, HRP-catalyzed polymerization of 3,3′-diaminobenzidine generated insoluble precipitates that significantly altered the optical thickness of the biosensor layer. This localized amplification enabled the highly sensitive, rapid, and on-site detection of PTX, a critical capability for acute poisoning investigations. Similarly, Tian et al. established a high-sensitivity colorimetric immunoassay for OA by integrating a dual-catalytic amplification strategy utilizing Au@Pt nanoparticles alongside HRP in a TMB-H_2_O_2_ indicator system [182]. Achieving an LOD of 0.04 ng/mL, this method represents a robust approach for ultra-trace OA profiling.

Oxidases catalyze the oxidation of specific substrates by utilizing molecular oxygen (O_2_) as a terminal electron acceptor. A prominent example is glucose oxidase (GOx), which catalyzes the oxidation of glucose to yield gluconic acid and H_2_O_2_. This in situ generation of H_2_O_2_ is frequently coupled with secondary signal transduction cascades. Tang et al. elegantly exploited this mechanism to develop a competitive photoelectrochemical immunoassay for OTA [183]. In this system, GOx-catalyzed H_2_O_2_ generation was utilized to trigger the localized chemical etching of silver nanoparticles (AgNPs). Integrated with an Ag@AgCl/reduced graphene oxide (RGO) photoactive heterostructure, this etching process modulated the photocurrent, achieving an exceptional LOD of 4.0 pg/mL (0.01 nM) and demonstrating excellent analytical performance for trace target analysis.

Alkaline phosphatase (ALP) is a hydrolase that catalyzes the dephosphorylation of phosphate esters into corresponding alcohols or phenols. In advanced biosensors, ALP-mediated substrate hydrolysis is highly effective when coupled with photoelectrochemical readouts. Su et al. utilized a competitive immunoassay format between target AFB_1_ and an AFB_1_-BSA-ALP conjugate [184]. Upon specific binding, the immobilized ALP catalyzed the hydrolysis of ascorbic acid 2-phosphate to generate ascorbic acid. Taking advantage of the specific chemical etching capability of ascorbic acid on cobalt oxyhydroxide CoOOH, the CoOOH/CdS surface layer was progressively degraded while the underlying CdS layer remained intact. This structural modulation generated an amplified photocurrent, offering a highly sensitive transduction pathway.

While biological enzymes have fundamentally driven the advancement of highly sensitive biotoxin detection, their inherent biochemical vulnerabilities restrict their universal application, particularly in demanding forensic environments. Natural enzymes suffer from prohibitive production costs, require stringent temperature-controlled storage, and are highly susceptible to rapid denaturation and loss of catalytic activity when exposed to extreme pH, temperature fluctuations, or the proteolytic enzymes frequently encountered in degraded postmortem matrices. To circumvent these critical bottlenecks, analytical chemists have persistently explored robust synthetic alternatives. Consequently, enzyme-mimicking nanomaterials (nanozymes) and catalytic oligonucleotides (DNAzymes) have emerged as powerful substitutes. By seamlessly integrating target recognition with highly durable, enzyme-like signal amplification, these intelligent synthetic strategies are garnering significant attention for the next generation of robust forensic biosensing.

### 4.2. Nanozymes

Nanozymes are a pioneering class of synthetic nanomaterials possessing intrinsic enzyme-mimicking catalytic activity. Compared to natural biological enzymes, nanozymes present several distinct analytical advantages critical for robust forensic applications: (1) extreme tolerance to harsh physicochemical environments (e.g., the extreme pH and elevated temperatures often required to extract toxicants from degraded medicolegal matrices); (2) highly tunable catalytic activity coupled with large specific surface areas, facilitating dense target-specific bio-functionalization; (3) unprecedented long-term shelf-stability and low-cost scalable production; and (4) the synergistic integration of unique secondary physicochemical properties, such as superparamagnetism, photothermal capabilities, or localized surface plasmon resonance (LSPR) [185]. Since Yan’s research group first demonstrated in 2007 that Fe_3_O_4_ magnetic nanoparticles exhibit intrinsic horseradish peroxidase (HRP)-like activity [186], nanozymes have emerged as highly durable alternatives to natural enzymes in biosensor development. To date, nanozymes synthesized from noble metals, cerium, manganese, and copper have been extensively leveraged for biotoxin detection [185,187]. This section explores their application in amplifying molecular recognition signals, focusing primarily on their peroxidase-like and oxidase-like activities within advanced forensic biosensors.

A prominent trend in toxicological sensing involves deploying nanozymes with intrinsic peroxidase-like (POD-like) activity to overcome the diffusion limitations of traditional assays. For example, POD-like nanozymes can simultaneously serve as high-density structural carriers for chemiluminescent (CL) substrates (e.g., luminol). This directly localizes the catalytic reaction at the sensor interface, preventing signal diffusion and dramatically amplifying the read-out. Yan et al. developed a highly sensitive CL biosensor for OTA utilizing AuNP nanozymes [188]. In this system, AuNPs modified with complementary DNA strands specifically recognized OTA aptamer-functionalized magnetic microspheres. Following rapid magnetic separation—a distinct advantage for isolating targets from viscous biological samples—the AuNPs catalyzed the luminol system. Demonstrating a dynamic range of 0.01–20 ng/mL and an exceptional LOD of 0.0067 ng/mL, this platform provides the ultra-sensitivity required for trace toxicological analysis. Similarly, Liu et al. employed Cu/Co bimetallic nanozymes labeled with MC-RR aptamers as robust signal-amplifying probes for highly sensitive affinity-based detection [189]. Addressing the critical need for multiplexed forensic screening, Jiang et al. leveraged the exceptional plasmonic properties of AgNP nanozymes to construct a high-throughput, plasmon-coupled electrochemical immunosensor [190]. By integrating carboxyl-functionalized AgNPs with biotoxin-coated antigens on an amino-modified glass chip, the optically active AgNPs enhanced the CL signal of HRP-labeled secondary antibodies via LSPR effects. Utilizing a charge-coupled device, this platform simultaneously captured amplified signals across multiple sites, enabling the concurrent profiling of diverse biotoxins from a single complex sample. 

Beyond POD-mimetic nanomaterials, several nanozyme classes exhibiting robust oxidase-like (OXD-like) activity have been strategically integrated into analytical diagnostics. Zhao et al. described a surface-enhanced Raman scattering (SERS) biosensor for AFB_1_ incorporating gold–mercury nanoparticles (Au@HgNPs) conjugated with carbon dots (CDs) [191]. The strategic introduction of Hg^2+^—subsequently reduced to metallic (Hg^0^) during nanoparticle formation—significantly ameliorated the colloidal instability and weak catalytic activity typical of unmodified AuNPs, facilitating robust oxidase-mimicking behavior. In the presence of AFB_1_, specific interactions with the toxin’s carbonyl oxygen atoms effectively suppressed the agglomeration of the Au@HgNPs, thereby maintaining a highly amplified and quantifiable SERS signal. In the context of rapid, on-site medicolegal investigations, Cai et al. developed an innovative lateral flow assay (LFA) utilizing MnO_2_ nanozymes as both an OXD-mimetic catalyst and a direct visual marker [192]. Utilizing the intrinsic capacity of MnO_2_ to oxidize TMB without requiring the addition of H_2_O_2_, the target biotoxin triggered a distinct visual colorimetric response on the test line. This nanozyme-enhanced test strip achieved an extraordinary LOD of 15 pg/mL for AFB_1,_ exceeding trace analytical requirements and providing sensitivity levels more than two orders of magnitude lower than standard regulatory thresholds.

Despite these profound analytical successes, the current paradigm of nanozyme-based biotoxin detection remains fundamentally constrained by its heavy reliance on peroxidase- and oxidase-like activities. Nanozymes exhibiting other critical catalytic functions—such as hydrolase, catalase, or multi-enzyme cascade mimicry—remain sparsely developed and underutilized in biosensor construction. Consequently, discovering novel nanozyme architectures with expanded, highly specific catalytic repertoires presents a significant hurdle, yet it represents a crucial frontier for engineering the next generation of highly intelligent, multi-modal forensic biosensors. 

### 4.3. DNAzymes

DNAzymes are catalytic, single-stranded DNA oligonucleotides isolated in vitro via SELEX that possess both high-specificity target recognition and highly efficient catalytic capabilities [193]. The foundational work by Breaker et al., demonstrating that short ssDNA molecules could catalyze the hydrolytic cleavage of RNA phosphodiester bonds, shattered the long-held paradigm that DNA was a structurally and functionally inert repository of genetic information [194]. Subsequently, a diverse array of DNAzymes with distinct catalytic activities—including nucleic acid cleavage, ligation, and phosphorylation—has been discovered. Compared to traditional protein enzymes, DNAzymes offer formidable advantages for trace analytical toxicology: (1) Exceptional thermodynamic stability: They retain catalytic activity even after exposure to high temperatures or the extreme acidic/alkaline conditions frequently required to extract toxicants from severely degraded medicolegal samples, allowing for robust storage and prolonged shelf-life. (2) Scalable synthesis: They are easily and cost-effectively chemically synthesized, exhibiting minimal batch-to-batch variability. (3) Cofactor versatility: Their catalytic activity is strictly dependent on specific cofactors (e.g., metal ions, small molecules, or target biotoxins), offering an intrinsic mechanism for target-triggered activation. (4) Structural programmability: Their flexible sequence design enables precise allosteric modifications and structural tuning. (5) Seamless amplification integration: Given their nucleic acid nature, DNAzymes seamlessly integrate with enzyme-free isothermal amplification technologies (e.g., HCR and RCA), enabling immense signal amplification without the need for specialized laboratory equipment. Consequently, DNAzymes serve as ideal molecular tools for developing highly sensitive, on-site forensic screening methodologies. 

Highlighting their practical utility, numerous DNAzyme-based aptasensors have been engineered for trace biotoxin profiling. A prevalent strategy involves the use of G-quadruplex sequences that bind hemin to form peroxidase-mimicking catalytic cores. For instance, Wang et al. and Setlem et al. developed distinct colorimetric quantification strategies for AFB_1_ utilizing peroxidase-mimicking DNAzymes [195,196]. Wang et al. designed a split-DNAzyme system that competes with an aptamer for AFB_1_ recognition. Conversely, Setlem et al. leveraged a closed-loop blocking sequence to sequester the DNAzyme. In this scheme, specific AFB_1_ interaction with the aptamer domain induces the structural unfolding of the ring, permitting DNAzyme assembly and subsequent colorimetric signal generation. Employing a target-protection mechanism, Yu et al. developed an exonuclease-mediated approach to detect OTA [197]. This methodology integrates an OTA aptamer with a peroxidase-mimetic DNAzyme sequence into a single-stranded entity. In the presence of OTA, the target binds the aptamer, sterically shielding the single strand from exonuclease degradation. The surviving sequence subsequently intercalates hemin to form an active catalytic core, generating a strong signal. In the absence of OTA, the exonuclease rapidly digests the unprotected single strand, permanently disabling signal generation.

Addressing the forensic challenge of complex mixed intoxication scenarios, Pan et al. developed a sophisticated multiplexed detection system based on CHA coupled with DNAzyme cleavage activity [198]. Under optimal conditions, this platform simultaneously detected OTA, AFB_1_, and ZEN with exceptional limits of detection of 0.2, 0.13, and 0.17 pM, respectively. Pushing the boundaries of intelligent diagnostics, the team further utilized these three biotoxins as molecular inputs (encoded as binary values 0 and 1) to construct a multi-serial molecular logic gate. By executing complex Boolean logical operations (e.g., “AND-INHIBIT”, “INHIBIT-OR”, “OR-AND”, and “OR-INHIBIT”), this programmable logic gate architecture provides a universal, highly adaptable sensing strategy for the simultaneous multiplex profiling of diverse toxicants in complex analytical matrices.

## 5. Recognition Molecules for Biotoxin Detection Across Biological Matrices

The selection of recognition molecules for biotoxin detection in forensic toxicology is fundamentally influenced by the biological matrix under investigation. Blood, urine, postmortem fluids, hair, nails, and alternative tissues each present distinct physicochemical characteristics that dictate which recognition systems perform optimally and what specific factors must guide their selection. This section examines the performance of antibodies, aptamers, and MIPs across different matrices, highlighting key considerations, limitations, and challenges for real-sample application.

### 5.1. Antibodies: Established Performance with Matrix-Specific Constraints

Antibodies remain the most extensively validated recognition elements in forensic toxicology, particularly when integrated with mass spectrometry workflows. Their performance across matrices has been well-characterized. For instance, polyclonal antibodies raised against abrin and ricin have been designed to enrich these highly toxic proteins from human blood and urine, enabling detection limits of at least 5 ng/mL in plasma when coupled with mass spectrometry [199]. This immuno-extraction approach has proven effective in complex matrices, including food and clinical specimens, enabling unambiguous differentiation of toxin isoforms and simultaneous quantification.

Antibody-based detection of biotoxins in blood, urine, and hair is constrained by matrix-specific limitations that compromise assay reliability. In blood, protein-rich environments, hemolysis, and postmortem decomposition interfere with antibody binding and generate false positives from putrefactive amines, while central blood is unsuitable for quantification due to postmortem redistribution [199]. In urine, antibodies perform excellently, making this matrix the gold standard for routine drug and toxin screening due to its high analyte concentrations and non-invasive collection; nevertheless, phase II metabolites (glucuronide or sulfate conjugates) require enzymatic hydrolysis prior to detection. This is primarily because these bulky, negatively charged conjugate groups introduce severe steric hindrance that prevents antibody binding to the core toxin skeleton. This necessitates either a pre-analytical enzymatic digestion step (using *β*-glucuronidase) to cleave the conjugate moiety, or the engineering of broad-spectrum antibodies capable of cross-reacting with both the parent toxin and its principal conjugated forms. Additionally, variations in pH and ionic strength can affect binding affinity [200]. In hair, rigorous decontamination is needed to distinguish external contamination from ingested compounds, harsh extraction conditions may degrade antibodies, and melanin-dependent incorporation rates preclude quantitative interpretation. Across all matrices, antibodies face persistent limitations including cross-reactivity with structurally similar compounds, batch-to-batch variability, susceptibility to denaturation under organic solvent conditions, inability to distinguish between isomers, and the necessity of confirmatory mass spectrometry, making antibody-based methods valuable as screening tools but insufficient as standalone forensic evidence [201].

### 5.2. Aptamers: Emerging Potential with Stability Advantages

Aptamers have demonstrated significant utility in detecting biotoxins across various biological matrices, leveraging their advantages of chemical synthesis, thermal stability, and cost-effectiveness. In serum and urine, aptamer-based assays have shown promising performance for detecting fungal toxins. A fluorescently labeled aptamer structure-switching assay developed for gliotoxin detection achieved a detection limit of 0.05 nM in both spiked serum and urine samples, with mean recoveries of 98.76–110.85%, demonstrating excellent selectivity and reproducibility [202]. For α-amanitin, the primary lethal toxin in Amanita mushrooms, a magnetic bead-based enzyme-linked immunoassay utilizing a DNA aptamer achieved detection limits of 0.337 µg/mL in urine and 0.372 µg/mL in mushroom samples, with desirable accuracy confirmed through recovery studies [203]. A UV-excitable aptasensor developed for aflatoxin B1 demonstrated recovery rates of 86.90–102.74% in crude brown sugar, processed peanuts, and long-grain rice, with results comparable to HPLC-PDA detection. This highlights aptamers’ potential for forensic toxicology in food matrices where antibody stability may be compromised [204].

Despite their promise, aptamer-based detection systems face significant challenges when applied to complex biological matrices. The most critical issue is matrix interference, which can severely compromise aptamer performance. A systematic investigation of tetrodotoxin detection in seafood matrices revealed that matrix components, particularly proteins and ionic strength variations, impaired aptamer structural stability and blocked binding sites, leading to 2.8 to 29.7-fold increases in detection limits compared to binding buffer [205]. This effect was attributed to the formation of aptamer–protein complexes that obstructed target binding, emphasizing that aptamers with stable conformations exhibit greater resistance to matrix interference. Another major concern is surface fouling and signal degradation in biological fluids. Studies on electrochemical aptamer-based sensors deployed in undiluted biological fluids at physiological temperatures determined that signal loss is primarily driven by desorption of monolayer components from the sensor surface rather than nuclease hydrolysis, limiting in vivo operational life to less than 12 h [206]. While L-DNA aptamers can confer nuclease resistance, the fundamental issue of passive monolayer loss must be addressed before long-term applications become feasible. Additionally, nucleic acid aptamers are susceptible to enzymatic degradation by nucleases in biological fluids, requiring chemical modifications that may alter binding dynamics and increase production complexity. These challenges highlight that while aptamers offer advantages over antibodies for certain applications—particularly for small-molecule targets and field-deployable detection—their performance in complex biological matrices remains highly dependent on careful assay design, matrix pretreatment, and structural optimization to overcome interference and stability limitations.

### 5.3. MIPs: Stability with Implementation Challenges

MIPs have emerged as robust synthetic receptors for biotoxin detection across various biological matrices, offering superior chemical stability and design flexibility compared to natural recognition elements such as antibodies. In serum and urine, MIPs have demonstrated significant potential for detecting highly toxic biotoxins. A β-cyclodextrin-collaborated MIP, using the common moiety of five amanita toxins as the template, achieved detection limits of 0.34–0.42 µg/L in serum, 0.16–0.33 µg/L in urine, and 0.035–0.056 µg/kg in liver samples when coupled with ultra-high-performance liquid chromatography tandem mass spectrometry, showing great potential for diagnosing mushroom poisoning [207]. In complex food matrices, MIPs have been extensively applied to mycotoxin detection. For example, MIP-based electrochemical sensors for OTA demonstrated a detection limit as low as 35 pg/mL in coffee, almond, corn, and pistachio samples with excellent reproducibility and stability [208]. MIPs have also been integrated into solid-phase extraction for aflatoxin preconcentration in cereal grains, dry nuts, oil seeds, and milk, achieving recoveries of 79.1–109.4% [209].

Despite their significant advantages, MIPs face several critical challenges that limit their translation from laboratory studies to practical, field-deployable applications. The most persistent issue is template leakage, where residual template molecules trapped within polymer cavities leach out during analysis, causing false-positive results and compromising quantitative applications, a critical concern for forensic toxicology where legal outcomes depend on analytical accuracy [210]. Another major challenge is nonspecific adsorption and matrix interference; the high surface area of MIPs can lead to interactions with abundant matrix components such as proteins and lipids in complex biological samples, reducing effective binding capacity and assay selectivity [211]. The presence of cross-reactivity with structurally similar compounds also remains a concern when screening for multiple mycotoxins or novel toxins. Reproducibility and scalability issues persist due to the lack of standardized protocols for MIP synthesis, characterization, and validation, making inter-laboratory comparability difficult. Additionally, the mechanism of molecular recognition in MIPs is still not completely understood, hindering rational design improvements. While MIPs offer excellent stability under extreme pH, temperature, and organic solvent conditions—making them advantageous for aggressive extraction protocols—their generally lower binding affinities compared to antibodies and the need for extensive optimization for each target analyte remain significant barriers. Future advancements will require interdisciplinary convergence of computational modeling, AI-guided MIP design, and sustainable nanomaterial engineering to overcome these limitations and enable robust, field-deployable biosensing platforms.

### 5.4. Extraction and Purification Strategies in Forensic Casework

Translating molecular recognition elements into practical forensic tools fundamentally necessitates robust extraction and purification strategies, as biotoxins are typically deeply embedded within highly chaotic matrices such as whole blood, urine, highly acidic gastric contents, putrefied postmortem fluids, or contaminated dietary vehicles. Conventional analytical gold standards, such as LC-MS/MS, strictly require tedious and time-consuming sample pretreatment to mitigate severe matrix effects before analysis can proceed.

In forensic scenarios, the direct application of natural biological recognition elements during sample extraction faces severe limitations. For instance, postmortem samples are frequently characterized by extreme pH fluctuations, high concentrations of proteolytic enzymes, and complex cellular debris, which rapidly cause the irreversible conformational denaturation and inactivation of natural antibodies [212]. To overcome these intense extraction barriers, synthetic and highly resilient recognition molecules are increasingly deployed. Molecularly imprinted polymers (MIPs) possess an extreme environmental tolerance that traditional biomacromolecules cannot match; they can withstand high temperatures, resist extreme acid or alkali corrosion, and remain entirely immune to the complex organic solvents typically used for toxin extraction [213]. This makes MIPs exceptionally robust solid-phase extraction sorbents for harsh medicolegal samples. Similarly, nucleic acid aptamers offer significant advantages during sample processing; their structural denaturation under extreme environmental temperatures or ionic strengths is entirely reversible upon returning to appropriate buffer conditions, greatly facilitating their use in aggressive extraction protocols.

Furthermore, the kinetic clearance of macromolecular toxins in biological matrices heavily influences the choice of target analytes during sample extraction. For instance, macromolecular proteinaceous biothreats like ricin are cleared rapidly from circulation and easily degraded [201]. To circumvent this, forensic extraction protocols frequently focus on isolating ricinine, a highly stable, low-molecular-weight alkaloid biomarker co-extracted from castor seeds and excreted intact in urine [214]. Targeting ricinine serves as a robust surrogate to confirm exposure in highly degraded postmortem casework specimens, where intact protein detection is no longer viable.

Recent advancements also highlight the integration of extraction mechanisms with advanced nanomaterials and miniaturized platforms. For example, specific gold nanorod arrays have been successfully utilized to achieve the highly selective extraction and simultaneous surface-enhanced Raman scattering (SERS) detection of trace aconitine directly from chaotic food residue matrices [215]. Finally, to streamline casework and minimize the biohazard risks associated with transferring highly lethal toxins, the future of forensic extraction lies in integrated miniaturization. Paper-based microfluidics (µPADs) and lab-on-a-chip (LOC) systems are successfully compressing sample pretreatment, isolation, and detection into single miniaturized devices, fundamentally rewriting the operational rules for portable toxicological analysis [216,217].

Beyond the selection of extraction methods and recognition elements, the physicochemical nature of the biological matrix strictly dictates the choice of signal labels. In optically complex or hemolyzed forensic matrices (e.g., putrefying blood and unpurified tissue homogenates), traditional colorimetric or fluorescent dyes frequently suffer from severe background absorption and autofluorescence, necessitating the use of electrochemical transducers or advanced labels like upconversion nanoparticles. More critically, in harsh environments laden with active proteases and extreme pH shifts, such as degraded postmortem fluids or highly acidic gastric contents, natural biological enzyme labels (e.g., HRP or ALP) undergo rapid, irreversible denaturation. As previously detailed in Section 4, these specific matrix constraints fundamentally drive the forensic preference for integrating recognition molecules with highly durable synthetic labels, particularly nanozymes and DNAzymes, which maintain robust signal amplification despite extreme medicolegal conditions.

### 5.5. Commercial Viability and Translational Hierarchy

From a commercialization and practical translation perspective, a clear adoption hierarchy exists among these molecular recognition elements. Currently, traditional antibodies completely dominate the commercial in vitro diagnostics (IVD) market [61]. However, as the demand for stability in extreme forensic matrices grows, nanobodies and aptamers represent the most commercially viable next-generation candidates. Nanobodies offer the high target specificity of traditional antibodies but with vastly superior thermal stability, allowing seamless integration into existing commercial ELISA manufacturing infrastructures. Aptamers closely follow, driven by their ultra-low-cost solid-phase chemical synthesis and their unique structural flexibility, which enables their recent integration with widely available commercial personal glucose meters (PGMs) for equipment-free point-of-care testing [218,219].

Conversely, synthetic peptides and MIPs currently lag significantly behind in broad commercial diagnostic adoption [220,221]. The commercial viability of synthetic peptides is primarily hindered by high initial discovery costs and generally lower binding affinities. Meanwhile, the commercialization of MIP-based sensors is heavily bottlenecked by unresolved issues with “template leakage”—which risks legally disastrous false-positive results in forensic casework—and a fundamental lack of standardized, large-scale synthesis protocols. Therefore, while MIPs are commercially valuable as solid-phase extraction (SPE) consumables, nanobodies and aptamers are undoubtedly the frontrunners for integration into next-generation commercial toxin detection sensors.

## 6. Conclusions and Future Perspectives

Biotoxins are highly potent toxicants that pose severe threats in cases of accidental, occupational, or intentional poisoning. Because they exhibit extreme toxicity at minute doses and are frequently encountered within highly complex biological matrices, their rapid and precise identification remains a paramount challenge in forensic and analytical toxicology. Gaining a thorough understanding of these molecular entities and establishing robust monitoring methodologies is crucial for determining causes of death, investigating acute exposures, and safeguarding public health. This comprehensive review has summarized recent advancements in biotoxin identification, detailing the structural characteristics, preparation methodologies, and analytical efficacy of specific binding agents and signal amplification strategies. Ultimately, the quality and resilience of the recognition molecule dictate the performance and reliability of any rapid detection technique.

Rather than relying on a one-size-fits-all approach, the selection of an optimal recognition molecule is now a highly strategic decision dictated by the target analyte and the physicochemical severity of the analytical matrix. Traditional and recombinant antibodies remain foundational due to their unparalleled initial avidity; however, their susceptibility to denaturation and proteolytic cleavage in harsh postmortem environments remains a critical bottleneck. Conversely, aptamers and affinity peptides offer highly programmable, synthetic alternatives with superior chemical resilience, though they frequently require structural stabilization (e.g., terminal capping or chemical backbone modifications) to combat nuclease degradation and matrix interference in raw biological and environmental samples. Addressing the extreme end of physicochemical stability, molecularly imprinted polymers (MIPs) provide remarkably durable, reusable synthetic cavities. While MIPs excel in extreme extraction conditions, ongoing challenges such as limited functional monomer diversity and the risk of “template bleeding” (leading to false positives) necessitate rigorous synthesis optimization. Moving forward, the field is transitioning from treating these platforms in isolation to exploiting their complementary strengths, often integrating them in dual-recognition hybrid assays to maximize both affinity and matrix tolerance.

Despite substantial progress in recognition element design, achieving the ultra-trace detection limits required by modern forensic standards increasingly relies on the parallel advancement of catalytic signal amplification. The strategic integration of specific recognition molecules with catalytic amplifiers is flourishing. Notably, DNAzymes and synthetic nanozymes offer enzyme-like catalytic performance combined with the extreme environmental stability required to withstand harsh toxicological sample processing. However, a significant analytical hurdle remains: the inherent catalytic turnover rates of many current artificial enzymes are frequently insufficient to outpace natural biological enzymes. To bridge this performance gap, future catalytic investigations must focus on atomic-level structural refinement, hetero-atom doping for nanozymes, and the rational design of multi-enzyme cascade systems to exponentially boost catalytic efficiency and signal transduction.

In light of these technological advancements and persisting analytical challenges, the future developmental trajectory of forensic biotoxin recognition will be propelled by three primary paradigm shifts:

(1) Universal Computational Rational Design: Historically reliant on empirical in vivo immunization or resource-intensive in vitro library screening, the discovery of recognition elements is undergoing a profound paradigm shift toward in silico rational design. Across all platforms, from using molecular dynamics (MD) simulations to predict aptamer folding and Density Functional Theory (DFT) to optimize MIP monomer selection, to deploying AI-driven molecular docking for peptide and recombinant antibody engineering, advanced computational modeling will become the universal standard. By theoretically mapping hydrogen bonds, hydrophobic effects, and thermodynamic binding energies at the atomic level prior to physical synthesis, researchers can radically accelerate assay development, eliminate cross-reactivity, and design hyper-specific receptors tailored for chaotic matrices.
(2) Robust Scalability and Standardization: Establishing reliable biotechnology and synthetic pipelines for the large-scale, standardized production of highly stable recognition molecules is imperative. Overcoming current translational bottlenecks—such as batch-to-batch variability and high production costs—will directly bridge the gap between academic proof-of-concept sensors and fully commercialized, legally defensible forensic diagnostic kits.
(3) Smart and Portable Integration: The ultimate translational goal involves integrating these advanced recognition and amplification elements into miniaturized, automated point-of-care (POC) and lab-on-a-chip (LOC) instruments. Combining multiplexed recognition arrays with portable readout devices (e.g., smartphone-based optics or miniaturized electrochemical workstations) will streamline operational procedures. This integration will successfully transition sophisticated toxicological methodologies from controlled, resource-heavy laboratory environments to routine, on-site medicolegal investigations, fundamentally transforming the landscape of practical biotoxin detection.
(4) Commercial Value and Tiered Workflow: While commercial ELISA kits offer rapid, high-throughput, and cost-effective screening for biotoxins, they function primarily as presumptive tools in forensic toxicology. Because they inevitably face cross-reactivity, batch inconsistency, and severe matrix interference in postmortem samples, they cannot stand alone as absolute legal evidence. Ultimately, integrating these commercial screening assays with downstream LC-MS/MS confirmation establishes a tiered analytical workflow, optimizing both operational efficiency and the rigorous legal defensibility required in modern medicolegal investigations.

## Figures and Tables

**Table 1:** Comparative features of specific binding agents (polyclonal, monoclonal, and recombinant antibodies, and affinity peptides) for biotoxin recognition [3,40,41].

Features	Polyclonal Antibodies	Monoclonal Antibodies	Recombinant Antibodies	Affinity Peptides
Affinity	High avidity; multi-epitope targeting.	Exceptionally high; single-epitope specific.	Tunable via in vitro maturation and library screening.	Moderate to high; in silico or in vitro derived.
Uniformity	Poor; high batch-to-batch variability.	Excellent; consistent physicochemical properties.	Excellent; strictly genetically defined.	Exceptional; precise chemical synthesis.
Cross reactivity	High risk of false positives.	Highly specific; minimal cross-reactivity.	Highly specific; reducible via negative screening.	Highly specific; precise structural recognition.
Antigen requirements	High-purity antigens for in vivo immunization.	Impure for immunization, but strictly pure for screening.	Pure antigens for in vitro library screening.	Pure for in vitro; none for in silico design.
Supply	Limited by animal lifespan and blood volume.	Unlimited in vitro proliferation via cell lines.	Unlimited scalable microbial/mammalian expression.	Unlimited and rapid solid-phase chemical synthesis.
Matrix tolerance	Poor; susceptible to denaturation in solvents.	Moderate; bulky size induces steric hindrance.	High; compact structure and superior stability.	Exceptional resilience to harsh extraction conditions.
Cost efficiency	Low overall production cost.	High initial and long-term production costs.	High setup cost; cost-effective long-term.	Variable discovery cost; very low production cost.

**Table 2:** Sequences of reported biotoxin aptamers.

Target	Sequence (From 5′ to 3′)	Length (nt)	Rounds	Type of SELEX	Kd (nmol/L)	Ref.
AFB_1_	CTCGTCTCGTTCTCTCAGTCGGACGAAGAGAGGGGGAGAGGGGGACGGAGCTGCTAAGGTGACACGAAGAAGAAGGAGGA	80	13	Affinity columns SELEX	85.02 ± 25.74	[108]
	AGCAGCACAGAGGTCAGATGTCTAAATGACACACTTTTCAACCTATCGACTTGGTTTACTACCTATGCGTGCTACCGTGAA	81	10	Magnetic bead SELEX	15.78 ± 2.47	[109]
AFB_2_	AGCAGCACAGAGGTCAGATGCTGACACCCTGGACCTTGGGATTCCGCGAAGTTTTCGGTACCTATGCGTGCTACCGTGAA	80	10	Magnetic bead SELEX	9.83 ± 0.99	[110]
AFM_1_	ATCCGTCACACCTGCTCTGACGCTGGGGTGCAGCCCGGAGAAATGCAATTCCCCTGTGGTGTTGGCTCCCGTAT	74	11	Magnetic bead SELEX	35.6 ± 2.9	[111]
OTA	TGGTGGCTGTAGGTCAGCATCTGATCGGTGTGGTGCGTTAAAGGGACATGTCGACAACG	59	13	Affinity columns SELEX	360	[112]
	CGGAGGACGAAGCGGACCCGGTCTGCGTGCCTTGATCCAGGGAGTCTCAGAAGACACGCCCGCACA	66	14	Magnetic bead SELEX	96	[113]
	AGCCTCGTCTCTTCTCCCGGCAGTGTGGGCGAATCTATGCGTCACCGTTCCAGATCCTGGCGAAGACAAGCAGACGT	77	15	Magnetic bead SELEX	290 ± 150	[114]
FB_1_	ATACCAGCTTATTCAATTCATCCAGTAACAAACACATAAGTAACGGCGATATGTCAAAGCGGTATCGGCTACAGATGAGATAGTAAGTGCAATCT	95	18	Magnetic bead SELEX	100 ± 30	[115]
	AGCAGCACAGAGGTCAGATGCGATCTGGATATTATTTTTGATACCCCTTGGGGAGACATCCTATGCGTGCTACCGTGAA	79	13	Magnetic bead SELEX	62 ± 5	[116]
OA	GGTCACCAACAACAGGGAGCGCTACGCGAAGGGTCAATGTGACGTCATGCGGATGTGTGG	60	N/A	Agarose bead SELEX	77	[117]
	ATTTGACCATGTCGAGGGAGACGCGCAGTCGCTACCACCT	40	13	Graphene oxide SELEX	40	[118]
GTX1/4	AACCTTTGGTCGGGCAAGGTAGGTT	25	8	Graphene oxide SELEX	17.7	[119]
MC-LR	GGCGCCAAACAGGACCACCATGACAATTACCCATACCACCTCATTATGCCCCATCTCCGC	60	14	Sepharose bead SELEX	50	[120]
MC-YR	CACGCAACAACACAACATGCCCAGCGCCTGGAACATATCCTATGAGTTAGTCCGCCCACA	60	14	Sepharose bead SELEX	28	
MC-LA	GGACAACATAGGAAAAAGGCTCTGCTACCGGATCCCTGTTGTATGGGCATATCTGTTGAT	60	14	Sepharose bead SELEX	193	
Patulin	CGAAATCGCGTCCAGTGTTGGGGCGTGCTTATCCTTACACGATTTACCTGAAACGCACCGTACTGAACTACGGCGAGGTC	80	N/A	Biolayer interferometry SELEX	82	[121]
STX	TAGGGAAGAGAAGGACATATGATGGCACAAGGCCTCATCAATCGGTATACGGGTTGACTAGTACATGACCACTTGA	76	9	Graphene oxide SELEX	50.8	[122]
	GGTATTGAGGGTCGCATCCCGTGGAACATGTTCATTGGGCGCACTCCGCTTTCTGTAGATGGCTCTAACTCTCCTCT	77	10	Magnetic bead SELEX	3840	[123]
T-2	CAGCTCAGAAGCTTGATCCTGTATATCAAGCATCGCGTGTTTACACATGCGACAGCTGAACACTCGAAGTCGTGCATCTG	80	10	Graphene oxide SELEX	20.8 ± 13.1	[124]

Abbreviations: AFB_1_, Aflatoxin B1; AFB_2_, Aflatoxin B2; AFM_1_, Aflatoxin M1; OTA, Ochratoxin A; FB_1_, Fumonisin B1; OA, Okadaic acid; GTX1/4, Gonyautoxin 1/4; MC-LR, Microcystin-LR; MC-YR, Microcystin-YR; MC-LA, Microcystin-LA; STX, Saxitoxin; T-2, T-2 toxin; nt, nucleotides; Kd, dissociation constant; SELEX, Systematic Evolution of Ligands by Exponential Enrichment.

**Table 3:** Characteristics of different preparation methods of MIPs.

Preparation Methods	Advantages	Disadvantages	Ref.
Bulk polymerization	Simple synthetic procedure; high overall density of recognition sites.	Mechanical grinding needed; irregular shapes; slow mass transfer.	[161]
Suspension polymerization	Regular spherical particles; adjustable size; protected cavities.	High dispersant usage; poor monodispersity; low surface area.	[162,163]
Emulsion polymerization	Uniform sub-micron spheres; excellent aqueous dispersibility.	Complex biphasic system; stringent optimization; surfactant interference.	[164,165]
Precipitation polymerization	One-step synthesis; surfactant-free; uniform & pure microspheres.	High solvent consumption; requires abundant high-purity templates.	[151]
Surface polymerization	Fast mass transfer; rapid equilibrium; fully exposed binding sites.	Complex substrate modification; lower overall site density.	[166,167]
Nano-imprinting polymerization	High specific surface area; ultra-fast response; controllable morphology.	Demanding conditions; prone to aggregation; potential specificity loss.	[168]
Click chemistry polymerization	Rapid kinetics; high yield; highly modular functionalization.	Complex monomer pre-synthesis; catalyst-induced cavity disruption.	[169]
Virtual imprinting polymerization	Eliminates template-bleeding; safe handling of toxic targets.	Challenging analog design; structural mismatches reduce affinity.	[170]

## Data Availability

No new data were created or analyzed in this study. Data sharing is not applicable to this article.

## Abbreviations

The following abbreviations are used in this manuscript:

MIP	Molecularly imprinted polymer
SELEX	Systematic Evolution of Ligands by Exponential Enrichment
MIT	Molecular imprinting technology
STX	Saxitoxin
TTX	Tetrodotoxin
AZA	Azaspiracid
DA	Domoic acid
ELISA	Enzyme-linked immunosorbent assay
mAbs	Monoclonal antibodies
PbTx	Brevetoxin
LOD	Limit of detection
OA	Okadaic acid
CTX	Ciguatoxin
scFv	Single-chain variable fragments
Fab	Antigen-binding fragments
Nb	Nanobodies
MC-LR/YR/LA	Microcystin-LR/YR/LA
FB	Fumonisin B
ZEN	Zearalenone
AFB	Aflatoxin B
AFM	Aflatoxin M
GTX	Gonyautoxin
SEB	*Staphylococcus aureus* enterotoxin B
OTA	Ochratoxin A
T-2	Trichothecenes
Aptasensor	Aptamer-based biosensor
ssDNA	Single-stranded DNA
PCR	Polymerase chain reaction
Mag-SELEX	Magnetic bead SELEX
GO-SELEX	Graphene Oxide SELEX
PAT	Patulin
RCA	Rolling circle amplification
HCR	Hybridization chain reaction
CHA	Catalytic hairpin assembly
HRP	Horseradish peroxidase
ALP	Alkaline Phosphatase
SERS	Surface-enhanced Raman scattering
POC	Point-of-care
